# Multimodal Patient-Specific Identification of Atrial Flutter Circuits From ECG Time Series Using Explainable Machine Learning

**DOI:** 10.1109/JTEHM.2026.3694616

**Published:** 2026-05-19

**Authors:** Samuel Ruipérez-Campillo, David Hernando, Elisa Ramírez, Sergio Castrejón, Cecilia Zapata, Carlos Rodríguez Carneiro, Julia E. Vogt, José Luis Merino, Francisco Castells, José Millet

**Affiliations:** Department of Computer ScienceETH Zürich 8092 Zürich Switzerland; Universitat Politècnica de València Valencia 46022 Spain; Department of CardiologyLa Paz University Hospital 16268 Madrid 28046 Spain

**Keywords:** Electrocardiography, explainability, medical machine learning, electrophysiology, vectorial analysis

## Abstract

Objective:Accurate pre-procedural identification of atrial flutter (AFL) mechanisms can streamline mapping and indirectly inform ablation strategy, yet surface-electrocardiogram (ECG) criteria remain unreliable and circuit definition is typically confirmed invasively.Methods and procedures:We analyzed 97 consecutive patients undergoing electrophysiological (EP) study with simultaneous 12-lead ECG and EP-verified AFL subtype; adenosine-induced atrioventricular AV block enabled extraction of clean atrial segments. We reconstructed atrial vectorcardiograms (VCGs) and engineered interpretable descriptors of loop morphology and kinematics, including archetype cosine correlation, geometric complexity, and velocity-based slow-occupancy indices, then fused these with clinical variables in an explainable tree-ensemble model evaluated with nested cross-validation.Results:VCG loops exhibited subtype-specific archetypes (within-class correlation: $0.832\pm 0.129$ CCCW, $0.874\pm 0.154$ CCW, $0.647\pm 0.127$ PMCCW, $0.667\pm 0.159$ PMCW; C: common; PM: perimitral; CW: clockwise; CCW: counter-CW). On the test set, the multimodal Random-Forest improved discrimination over VCG-only and clinical-only baselines, achieving AUROC of 0.870 (CCCW), 0.900 (CCW), 0.840 (PMCCW), and 0.790 (PMCW), with high sensitivity for common AFL (0.833 and 0.929) and very high specificity for PMCW (0.988).Conclusion:This interpretable framework provides a practical route to non-invasive, mechanism-oriented AFL stratification to support targeted mapping and more efficient ablation planning. Future work will focus on multicenter prospective validation and robust atrial-signal extraction without adenosine to broaden routine applicability.

Clinical and Translational Impact Statement: Clinical Research. An interpretable ECG+clinical model predicts AFL mechanism pre-procedure, guiding mapping and ablation strategy. Integration: export 12-lead ECG, compute VCG features, and display subtype probability with key drivers in the EP lab.

## I. Introduction

Cardiac arrhythmias impose a substantial global health burden, with significant contributions to morbidity, mortality, and healthcare costs [1]. Among atrial arrhythmias, atrial flutter (AFL) is second in prevalence only surpassed by atrial fibrillation (AF), with they both sharing risk factors and complications [2], [3]. AFL can impair quality of life and confer thromboembolic risk, underscoring the need for accurate diagnosis and timely management [2]. Catheter ablation offers a durable, often curative therapy, particularly for typical cavotricuspid isthmus (CTI)-dependent AFL, where acute success rates are high [4], [5].

Mechanistically, AFLs are caused by a reentrant circuit, being typical (or common) AFL its most frequent form, which encompasses a counterclockwise (CCW) right-atrial circuit that encircles the tricuspid annulus and traverse the CTI, producing a distinct sawtooth-like waveform on the 12-lead electrocardiogram (ECG) [6]. CTI-dependent circuits also comprise clockwise (CW) rotation, although it describes a dissimilar sawtooth-like waveform on the 12-lead ECG [7]. Beyond these forms, atypical AFL includes left-atrial macro–reentry—most notably PM circuits with CCW or CW rotation around the mitral annulus—as well as other scar-related circuits [4], [5]. Moreover, right-atrial variants (e.g. upper- or lower-loop reentry, scar-relted AFL) and left AFL after AF ablation further broaden the spectrum and complicate non-invasive discrimination [5], [8].

Identification of the underlying circuit (CTI-dependent vs. left-atrium reentry) has direct procedural and prognostic implications, whether determined invasively or estimated non-invasively before the procedure. For instance, distinguishing typical CTI-dependent flutter from PM flutter informs lesion strategy (CTI line vs. mitral isthmus line), anticipates procedure complexity, and may influence recurrence risk [4], [9]. Conversely, misclassification can lead to ineffective ablation, prolonged procedures, and repeat interventions [5].

Invasive electrophysiology (EP) studies and mapping remain the gold standard for confirming the circuit location and rotational direction, including via entrainment mapping [9], [10]. However, invasive mapping is resource-intensive and not without risk. Standard 12-lead ECG criteria (e.g. flutter-wave morphology, axis, and lead polarity) provide valuable clues, yet their reliability is limited by overlapping patterns, conduction abnormalities, and interobserver variability, making differentiating AFL forms notably challenging from the ECG alone [7], [11], [12]. Atrial activity is strongly masked by ventricular depolarization and repolarization, and while signal-processing techniques such as ventricular cancellation can improve atrial wave retrieval in AF [13], these methods remain ineffective in AFL due to temporal coupling between atrial and ventricular electrical activity.

Several non-invasive avenues have been explored to address these limitations. Transforming 12-lead ECGs into vectorcardiograms (VCGs) yields three-dimensional loops whose geometry and temporal evolution can encode rotational direction and circuit characteristics [14], [15]. High-density body-surface mapping and ECG imaging (ECGI) can localize atrial reentry with higher resolution, but they require specialized hardware/software and remain limited in availability [16], [17]. Nevertheless, previous studies frequently relied on limited and complex data, where ground truth is challenging [18], while undervaluing clinical variables that strongly correlate with specific mechanisms (e.g., PM flutter after AF ablation) [14], [19], [20]. In parallel, machine learning (ML) directly applied to standard ECGs has achieved strong performance across diverse arrhythmia tasks [21], motivating data-driven AFL subtype classification from conventional leads [22], [23]. Furthermore, to support clinical adoption, models should be interpretable at the patient level, clarifying how ECG/VCG and clinical features jointly drive predictions.

In this work, we address this problem by proposing a multimodal approach to non-invasive AFL subtype classification that fuses standard 12-lead ECG with readily available clinical data. Specifically, we target four clinically relevant classes:common (CTI-dependent) CCW, common (CTI-dependent) CW, PM CCW, and PM CW; reflecting practical ablation strategies and decision points [4], [5], [19]. From the ECG, we reconstruct per-cycle vector loops via an inverse Dower–type transform and compute quantitative descriptors of loop shape, orientation, archetype similarity, and kinematics that capture putative slow-conduction behavior [14], [24]. We integrate these with demographic and clinical variables associated with circuit propensity to enhance discriminative power and enable patient-specific predictions. Although the IDT was originally derived from a standard torso model optimized for QRST loops, it remains the most widely adopted transform for atrial VCG analysis in AFL [14], [15], [25], and AF [26],; alternative approaches such as the Kors regression method [27], [28], P-wave–optimized matrices [29], and PCA-based derivations [24] have been proposed but carry their own limitations and have not been validated for flutter waves [30], [31].

To balance performance with interpretability, we adopt tree-based ensemble learners and provide post-hoc, instance-level explanations. Our contributions are threefold: (i) a principled ECG-to-VCG representation with mechanistically motivated features for AFL; (ii) a multimodal, clinician-facing model that quantifies the joint influence of VCG and clinical variables on patient-level predictions; and (iii) an open, reproducible pipeline aligned with FAIR principles [32] to catalyze validation and translation in broader clinical settings. Collectively, these advances aim to improve non-invasive discrimination of AFL mechanisms, reduce reliance on purely invasive diagnosis for mechanism identification, and streamline ablation planning.

## II. Clinical Methods

After institutional approval (Comité de Ética de la Investigación; Hospital Universitario La Paz; HULP code: PI-3247), we enrolled 97 consecutive patients with AFL who underwent catheter-based EP intervention. Patients with complex corrected or uncorrected congenital cardiopathy were not included. Surface ECG and intracardiac electrograms (EGM) were acquired concurrently using a polygraph system (Labsystem Pro, Bard, Boston Scientific, USA) at a sampling frequency of 1 kHz. Signals generated by the electroanatomical navigation system during the procedure were exported and temporally aligned with the polygraph recordings to permit joint analysis and establish the EP reference standard.

**FIGURE 1.**
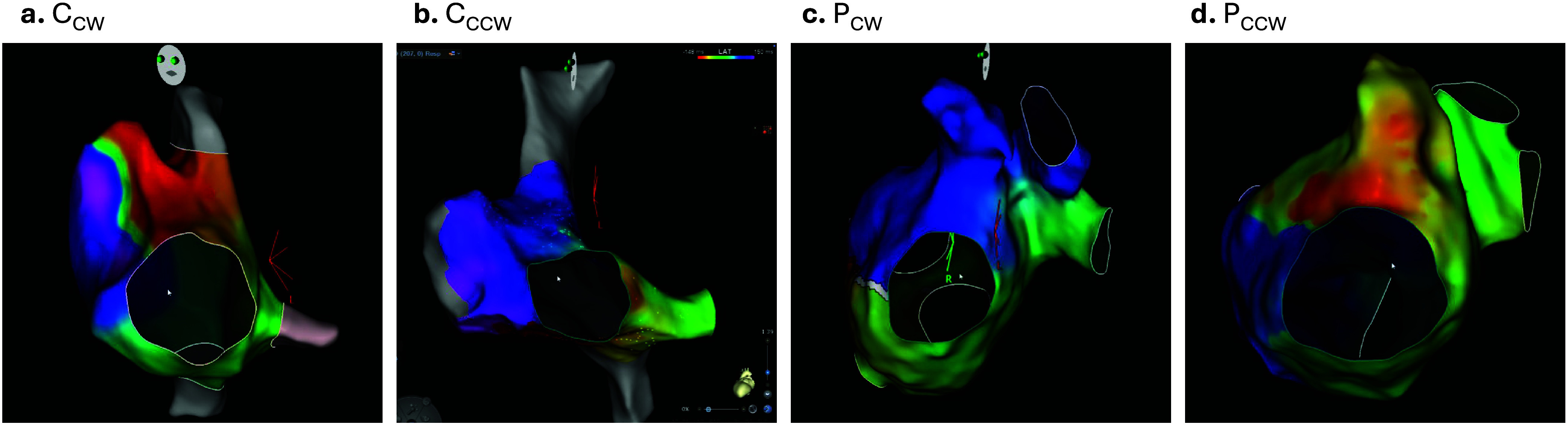
Representative electroanatomical activation maps illustrating one example of each macroreentrant circuit: (a) common CTI-dependent clockwise (C_CW_), shown in a 60-year-old male patient with no hypertension (BMI 34.78 kg/m^2^, LVEF 40%), no prior AF, and no previous left-atrial ablation; (b) common CTI-dependent counterclockwise (C_CCW_), shown in a 62-year-old male patient with hypertension (BMI 22.65 kg/m^2^, LVEF 60%), prior persistent AF, and previous left-atrial ablation; (c) perimitral (PM) clockwise (PM_CW_), shown in an 85-year-old female patient with hypertension (BMI 28.25 kg/m^2^, LVEF 65%), prior paroxysmal AF, and no previous left-atrial ablation; and (d) PM counterclockwise (PM_CCW_), shown in a 74-year-old female patient with hypertension (BMI 40.51 kg/m^2^, LVEF 60%), prior paroxysmal AF, and previous left-atrial ablation. In each panel, the color-coded activation sequence depicts the direction of propagation (red $\rightarrow $ orange $\rightarrow $ yellow $\rightarrow $ green $\rightarrow $ blue $\rightarrow $ indigo $\rightarrow $ violet) around the relevant annulus, while the region highlighted in red marks the slow-conduction zone sustaining reentry (see supplementary videos for the corresponding activation movies). This figure was generated using CARTO^®^ (Biosense Webster, USA).

During ECG acquisition, adenosine was administered to transiently suppress atrioventricular conduction, thereby isolating atrial activity and removing any ventricular contamination. Following completion of the EP study, the reentrant circuit was identified, ablated, and used to assign the AFL type as the ground-truth. Patients were then classified into four groups according to circuit location and rotation: common flutter clockwise (C_CW_, $n=30$), common flutter counterclockwise (C_CCW_, $n=36$), PM clockwise (PM_CW_, $n=12$), and PM counterclockwise (PM_CCW_, $n=19$). An electroanatomical activation map from a PM AFL is depicted in Figure 1, and the characteristics of the population are displayed in table 1. Echocardiographic variables (LAVi, RA area, E/e’, MR, TR) were available for a subset of patients as echocardiography was not part of the standardized EP protocol; n available is indicated for incomplete variables.

**TABLE 1:** Clinical Characteristics of the Study Population

Variable	AFL subtype				All
	C_CW_	C_CCW_	PM_CW_	PM_CCW_	
N	30	36	12	19	97
Age (years)	$72.5~\pm ~15.8$	$73.0~\pm ~9.4$	$67.3~\pm ~16.1$	$68.4~\pm ~10.5$	$71.2~\pm ~12.8$
Sex (women)	7 (23.3%)	9 (25.0%)	3 (25.0%)	5 (26.3%)	24 (24.7%)
HTA	20 (66.7%)	23 (63.9%)	6 (50.0%)	10 (52.6%)	59 (60.1%)
BMI (kg/m^2^)	$19.6~\pm ~13.4$	$25.9~\pm ~9.1$	$25.0~\pm ~8.7$	$26.7~\pm ~8.1$	$24.0~\pm ~10.7$
Height (cm)	$169.5~\pm ~7.9$	$169.3~\pm ~8.9$	$169.5~\pm ~10.9$	$167.8~\pm ~9.3$	$169.1~\pm ~8.8$
Weight (kg)	$70.0~\pm ~21.5$	$81.0~\pm ~15.6$	$78.2~\pm ~15.3$	$77.9~\pm ~21.6$	$76.7~\pm ~19.1$
EF (%)	$50.5~\pm ~13.5$	$51.2~\pm ~13.3$	$53.2~\pm ~9.9$	$53.4~\pm ~11.7$	$51.7~\pm ~12.6$
LAVi (ml/m^2^)	$38.6~\pm ~10.0$ (n=21)	$44.6~\pm ~13.4$ (n=26)	$46.4~\pm ~12.2$ (n=9)	$45.7~\pm ~17.7$ (n=14)	$43.3~\pm ~13.4$ (n=70)
RA area (cm^2^)	$20.3~\pm ~4.1$ (n=21)	$20.2~\pm ~5.1$ (n=26)	$20.3~\pm ~5.6$ (n=11)	$19.7~\pm ~4.4$ (n=15)	$20.1~\pm ~4.7$ (n=73)
E/e’	$10.4~\pm ~4.6$ (n=19)	$12.5~\pm ~5.2$ (n=22)	$10.3~\pm ~3.4$ (n=10)	$9.1~\pm ~2.6$ (n=12)	$10.9~\pm ~4.5$ (n=63)
MR $\geq $ moderate	3/24 (12.5%)	4/27 (14.8%)	2/10 (20.0%)	3/15 (20.0%)	12/76 (15.8%)
TR $\geq $ moderate	3/24 (12.5%)	3/27 (11.1%)	0/10 (0.0%)	0/15 (0.0%)	6/76 (7.9%)
Prior AF (parox)	6 (20.0%)	6 (16.7%)	5 (41.7%)	5 (26.3%)	22 (22.7%)
Prior AF (pers)	0 (0.0%)	7 (19.4%)	6 (50.0%)	9 (47.4%)	22 (22.7%)

Notes: Values are mean ±SD or $n(\%)$. Abbreviations: $\mathrm{HTA}=$ Hypertension; $\mathrm{BMI}=$ Body Mass Index; $\mathrm{EF}=$ Ejection Fraction; $\mathrm{AF}=$ Atrial Fibrillation; parox = paroxysmal; pers = persistent; C=Common; PM = Perimitral; CCW = counterclockwise; CW = clockwise. LAVi = indexed left atrial volume ($\mathrm{ml} / \mathrm{m}^2$); RA area = right atrial area $\left(\mathrm{cm}^2\right) ; \mathrm{E} / \mathrm{e}^{\prime}=$ diastolic dysfunction ratio; MR severity = mitral regurgitation (graded 0-3); TR severity = tricuspid regurgitation (graded 0-3).

## III. Technical Methods

**FIGURE 2.**
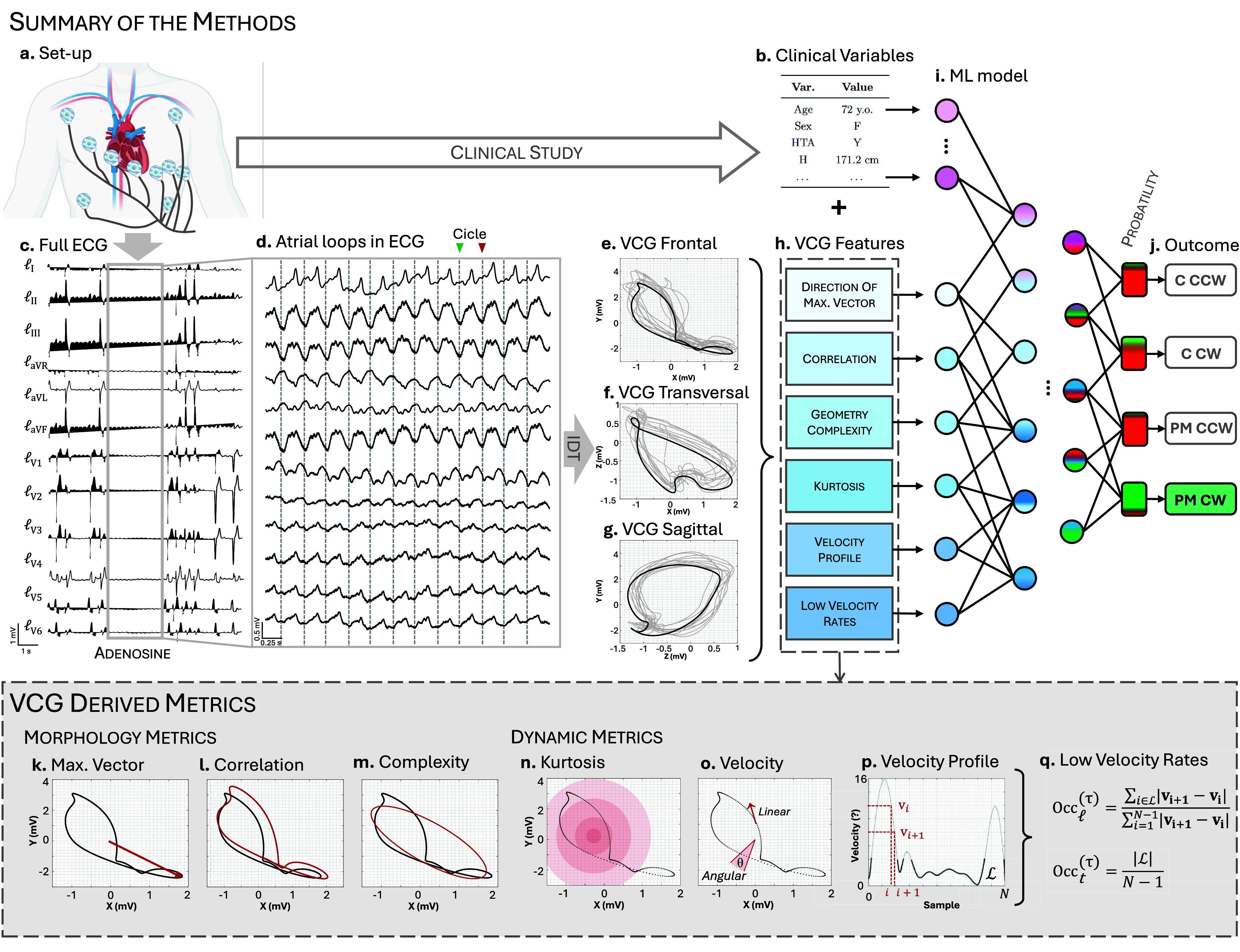
**Summary of the methods.** (a) Twelve-lead ECG electrodes, (b) patient-level clinical variables, (c) identification of the adenosine segment with stable atrial activity, (d) expert manual marking of consecutive atrial cycles, (e-g) reconstruction of the atrial VCG on frontal, sagittal, and transverse planes (h) Schematic overview of the extracted VCG-derived features including, (i) ML model schematic to a final probabilistic output, and (j) redicted outcome (AFL subtype). Features include morphology metrics: (k) maximum vector magnitude and direction on the VCG loop, (l) shape similarity (cosine correlation) with archetypal subtype loops, (m) geometric complexity; and dynamic metrics: (n) kurtosis, (o) velocity outline, (p) velicity profile, and (q) low velocity rates.

A summary of the methods is illustrated in Figure 2, and the code of this manuscript is reported in https://github.com/SamuelRuiperezCampillo/S_Ruiperez-Campillo_et_al_IEEE-JETHM_Multimodal_Patient-specific_AFL_from_ECG

### A. ECG Preprocessing

Twelve-lead ECGs (standard limb and precordial leads) were acquired at a sampling frequency $f_{s}$ of 1000 Hz. To enhance signal quality while preserving atrial morphology, a three-stage filter chain was applied uniformly to all leads: (i) a third-order Butterworth high-pass filter with 1.5 Hz cutoff to suppress baseline drift and respiratory components; (ii) an eighth-order Chebyshev type-II low-pass filter with 30 Hz cutoff to attenuate high-frequency noise; and (iii) a median filter with a 400-sample window (400 ms at $f_{s}=1000$ Hz) to further stabilize residual low-frequency excursions without distorting clinically relevant waveforms. For residual power-line interference, a 50 Hz notch filter was applied.

### B. Identification and Extraction of Atrial Segments

The analysis focused on the P-loop described by the VCG. During invasive testing, adenosine was administered by cardiologists to induce transient AV nodal block; this suppressed ventricular activity for a few seconds, thereby facilitating isolation of atrial activity in the surface ECG. On these segments, a panel of experts visually scanned lead II to locate the interval with maximal and most stable atrial activity (prominent F-waves), then extracted the corresponding multi-lead window synchronously across all 12 leads. For each patient, multiple atrial cycles within the selected interval were aligned and averaged to form a representative loop used for feature extraction and modeling; at least 3 consecutive cycles were confirmed per patient (typically $\approx 10$). A schematic of the atrial activity selection from ECG is shown in Figure 2.a (the 12-lead ECG with dashed boxes indicating the selected cycles), Figure 2.c (selection of section in adenosine for a recording), and Figure 2.d (manual marking of consecutive atrial loops by experts).

### C. Vectorcardiogram (VCG) Calculation

Let $\mathbf {e}(t)=[I,{\,}II,{\,}V_{1},{\,}V_{2},{\,}V_{3},{\,}V_{4},{\,}V_{5},{\,}V_{6}]^{\top } $ denote the ECG lead vector. The atrial VCG $\mathbf {r}(t)=[x(t),y(t),z(t)]^{\top } $ was reconstructed using the inverse Dower transform (IDT), $\mathbf {r}(t)=\mathbf {D}^{-1}_{0}{\,}\mathbf {e}(t)$, where $\mathbf {D}^{-1}_{0}\in \mathbb {R}^{3\times 8}$ is the standard inverse Dower matrix as in [33]. The 3D atrial loop was visualized and further analyzed in the frontal, sagittal, and transverse planes after projection (see Figure 2.e-f).

### D. Feature Extraction From the VCG

Let the resampled atrial loop be the discrete sequence $\{\mathbf {r}_{i}\}_{i=1}^{N}$ with $\mathbf {r}_{i}=[x_{i},y_{i},z_{i}]^{\top } $, spanning one atrial cycle. We define the following features that characterize the VCG **r** as follows (see Figure 2.k-q).

#### 1) Maximum Vector (Magnitude and Direction)

We identified the time index of maximum dipole magnitude,

\begin{align*} i^{\ast }=\arg \max _{1\le i\le N}\|\mathbf {r}_{i}\|_{2},\quad \mathbf {r}_{\max }=\mathbf {r}_{i^{\ast }},\quad ~m_{\max }=\|\mathbf {r}_{\max }\|_{2}, \tag {1}\end{align*}

and reported its orientation using spherical angles $(\theta,\phi)$, with $\theta =\arccos \!\big (z_{i^{\ast } }/m_{\max }\big)$ and $\phi =\mathrm {atan2}(y_{i^{\ast } },x_{i^{\ast } })$. The maximum vector summarizes the direction of the largest atrial activation vector on the VCG loop (see Figure 2.k).

#### 2) Geometric Complexity

To quantify morphological irregularity, we defined the *geometric complexity* as the ratio between the loop perimeter and the perimeter of a fitted ellipse:

\begin{equation*} \mathrm {Complexity}=\frac {P_{\mathrm {VCG}}}{P_{\mathrm {ellipse}}}. \tag {2}\end{equation*}

After projecting the loop onto the dominant 2D plane via PCA, the loop perimeter was computed discretely as $P_{\mathrm {VCG}}=\sum _{i=1}^{N-1}\big \|\tilde {\mathbf {r}}_{i+1}-\tilde {\mathbf {r}}_{i}\big \|_{2}$, with $\tilde {\mathbf {r}}_{i}\in \mathbb {R}^{2}$ denoting projected samples. An ellipse was least-squares fitted to $\{\tilde {\mathbf {r}}_{i}\}$ to obtain semi-axes $(a,b)$, and its perimeter was approximated [34] as $P_{\mathrm {ellipse}}\approx \pi (a+b)\!\left [{ 1+\frac {3h}{10+\sqrt {4-3h}}}\right]$ with $h=\left ({ \frac {a-b}{a+b}}\right)^{2}$. Values closer to 1 indicate an almost elliptical loop; values > 1 reflect increasing irregularity within the fitted ellipse (see Figure 2.m). We note that the first two principal components captured over 73% of loop variance for common AFL subtypes and over 68% for PM CCW, with the third component accounting for less than 10% in these classes (Supplementary table S.VIII). For PM CW, the third component reached $\approx 13\%$, consistent with the greater morphological heterogeneity of this subtype (see Section V). Although we retain the 2D-projected complexity for interpretability, a 3D generalization—where $\tilde {r}_{i} \in \mathbb {R}^{3}$ and the ellipsoid perimeter replaces the ellipse perimeter—could be explored in future work to better capture out-of-plane deviations, particularly for left-atrial circuits with complex anatomy.

#### 3) Shape Correlation With Archetypal Loops

For each AFL subtype, an archetypal loop was constructed by averaging normalized patient loops. The arithmetic mean was used to define a class-level reference loop rather than a single exemplar, ensuring that all patients contributed to the template after temporal alignment and normalization; this choice does not require Gaussian residuals, as the archetype serves only as a directional reference for cosine correlation scoring and is not used for distributional inference.. Using a train set to create the archetype, the test patient’s loop were correlated with all subtype archetypes. The predicted class corresponded to the highest correlation. Given matrices of normalized 2D samples $\tilde {\mathbf {R}},\hat {\mathbf {R}}_{k}\in \mathbb {R}^{N\times 2}$ (patient vs. $k$-th archetype), the cosine correlation $\rho _{k}$ was computed as

\begin{equation*} \rho _{k}=\frac {\langle \mathrm {vec}(\tilde {\mathbf {R}}),{\,}\mathrm {vec}(\hat {\mathbf {R}}_{k})\rangle }{\|\mathrm {vec}(\tilde {\mathbf {R}})\|_{2}{\,}\|\mathrm {vec}(\hat {\mathbf {R}}_{k})\|_{2}}{\,}, \tag {3}\end{equation*}

hence providing a compact, interpretable morphological similarity index (see Figure 2.l). To avoid data leakage, archetypes were recomputed independently within each training split of the nested cross-validation procedure and were never estimated using patients from the corresponding validation or test fold.

#### 4) Dynamic Metrics: Linear and Angular Kinematics

Instantaneous linear speed was computed from consecutive 3D samples,

\begin{equation*} v_{i}=\|\mathbf {r}_{i+1}-\mathbf {r}_{i}\|_{2} \tag {4}\end{equation*}

with $i=1, {\dots },N-1$, yielding the profile $\mathbf {v}=[v_{1}, {\dots },v_{N-1}]^{\top } $. To capture directional changes, we quantified the 3D turning angle between successive vectors and defined the discrete angular velocity as $\theta _{i}=\arccos \!\left ({ \frac {\mathbf {r}_{i}\cdot \mathbf {r}_{i+1}}{\|\mathbf {r}_{i}\|_{2}{\,}\|\mathbf {r}_{i+1}\|_{2}}}\right)$, and $\omega _{i}=\frac {\theta _{i}}{\Delta t}$, with $\Delta t=1/f_{s}^{\ast } $ the sampling interval of the resampled loop. In addition to summary statistics (mean, skewness, kurtosis) computed over $\{v_{i}\}$ and $\{\omega _{i}\}$, we calculated the area under the angular-velocity curve (AUC${}_{\omega } $) as a discriminative descriptor across AFL subtypes (see Figure 2.n-p).

#### 5) Slow-Velocity Percentages as Subthreshold Occupancy in the Velocity Profile

To probe regions of putative slow conduction, we thresholded the speed profile at a relative level $\tau =0.25$ of the loop’s maximum speed $v_{\max }$ and defined the index set $\mathcal {L}=\{i:\ v_{i}\le \tau {\,}v_{\max }\}$. This value was informed by empirical observations that non-invasive VCG correlates of slow-conduction regions consistently fall within the lower quartile of the velocity distribution across AFL types [14]. Because $\tau $ is defined relative to each patient’s own maximum speed, the descriptor remains comparable across patients with different loop amplitudes and cycle lengths. This threshold serves as a feature-construction parameter; the downstream classifier learns its own optimal decision boundaries over the resulting occupancy values. We then reported the *temporal subthreshold occupancy*,

\begin{equation*} \mathrm {Occ}_{t}^{(\tau)}=\frac {|\mathcal {L}|}{N-1}, \tag {5}\end{equation*}

and the *arc-length subthreshold occupancy*,

\begin{equation*} \mathrm {Occ}_{\ell }^{(\tau)}=\frac {\sum _{i\in \mathcal {L}}\|\mathbf {v}_{i+1}-\mathbf {v}_{i}\|_{2}}{\sum _{i=1}^{N-1}\|\mathbf {v}_{i+1}-\mathbf {v}_{i}\|_{2}}, \tag {6}\end{equation*}

which jointly summarize the temporal and spatial occupancy of slow-velocity zones on the loop.

We also summarize their relationship via $R^{(\tau)}=\mathrm {Occ}_{t}^{(\tau)}/\mathrm {Occ}_{\ell } ^{(\tau)}$ (see Figure 2.q).

#### 6) Within-Class Homogeneity

To quantify within-class morphological homogeneity, we performed PCA on the matrix stacking one representative loop per patient in a class and reported the fraction of variance explained by the first principal component,

\begin{equation*} \mathrm {C_{1}}=\frac {\lambda _{1}}{\sum _{j}\lambda _{j}}, \tag {7}\end{equation*}

as an intraclass consistency index (higher values indicate stronger alignment among loops of the same subtype).

### E. Statistical Analysis

Continuous variables are reported as mean±SD and/or median [IQR], as appropriate, while categorical variables are summarized as $n$ (%). Prior to hypothesis testing, the distributional assumption was assessed using the Kolmogorov–Smirnov test at a type-I error level of $\alpha =0.05$. As normality was not supported for the study variables, between-group comparisons were performed using non-parametric methods. Specifically, we used the Wilcoxon rank-sum test (equivalent to the Mann–Whitney $U$ test) to compare two independent groups without assuming Gaussianity; this approach also accommodates unequal sample sizes across AFL subtypes. Although omnibus non-parametric tests for comparing more than two groups (e.g., Kruskal–Wallis) are available, we adopted a pairwise comparison strategy to identify subtype-discriminative variables. When multiple pairwise contrasts were conducted across AFL subtypes, $p$-values were adjusted for multiple testing using Bonferroni correction. Statistical significance was defined as $p< 0.05$.

### F. Machine-Learning Modeling

**FIGURE 3.**
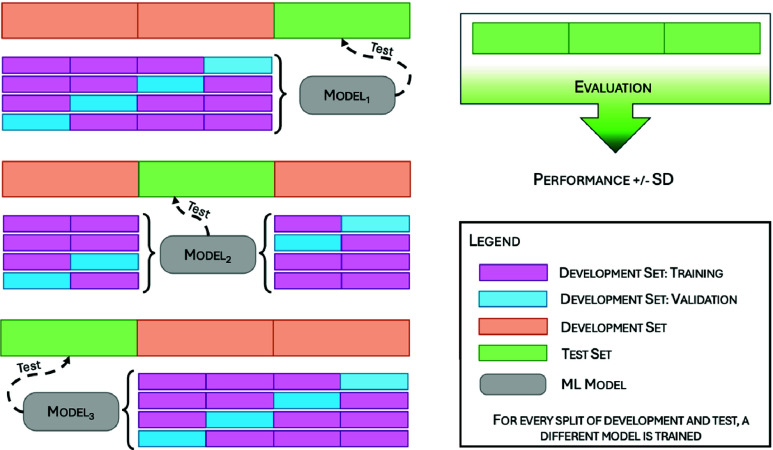
**Nested cross-validation scheme.** Each outer-fold iteration defines a development set (orange) that is split into training (purple) and validation (blue) folds in the inner loop for hyperparameter tuning and model selection. For each outer iteration, the selected hyperparameters are retrained on the full outer development set and evaluated once on the held-out unseen outer test set (green), yielding an unbiased estimate of generalization performance across folds.

Prior to training, continuous predictors were standardized (z-score) using parameters computed on the inner training folds only; categorical variables were one-hot encoded. The learning algorithms included an interpretable decision tree, a Random Forest (bagging ensemble), and AdaBoost. Tree-based ensemble learners were selected not only for their classification performance but also for their native interpretability properties. Random Forests provide impurity-based feature importance—computed from the weighted decrease in node impurity (Gini index) averaged across all rees—which directly quantifies the contribution of each VCG-derived and clinical feature to the classification. This form of global interpretability reflects the model’s internal split structure, though it can be biased toward high-cardinality continuous features and may dilute importance across correlated predictors [35]. Permutation importance (Supplementary figure S.I) was computed as a complementary measure that is less susceptible to these biases. Because each tree partitions the feature space via axis-aligned splits, the resulting importance rankings are directly traceable to specific features and thresholds, enabling clinicians to understand which combination of archetype correlations, kinematic descriptors, and clinical variables drives the prediction for a given patient. To assess overfitting risk given the modest sample size, two regularized linear baselines—Elastic Net logistic regression and a linear-kernel SVM—were included alongside the tree-based models in the nested cross-validation framework (Supplementary table S.IX). Nested cross-validation was used to obtain unbiased performance estimates: an outer $K$-fold loop provided external test sets; within each outer training set, an inner loop performed grid search over hyperparameters; the best inner configuration was retrained on the outer-training data and evaluated once on the held-out outer-test fold. For each outer fold, the same training and held-out test partition was used for all candidate models; likewise, within the inner loop, all models were compared on the same validation folds, ensuring an unbiased model-family comparison. A schematic of the nested cross-validation procedure is provided in Figure 3. Random Forest hyperparameters were tuned within the inner loop of nested cross-validation using grid search, and the final configuration was selected as the one achieving the best mean inner-validation macro-F1. The same grid-search procedure was applied to all candidate model families. The searched hyperparameter spaces are detailed in Supplementary Table S.VII. Representative outer-fold hyperparameters for the Random Forest are summarized in supplementary table S.VII. Model performance was quantified using overall accuracy, sensitivity, specificity, macro-F1, and one-vs-rest ROC–AUC. For a positive class,

\begin{equation*} \mathrm {F1}=\frac {2{\,}\mathrm {TP}}{2{\,}\mathrm {TP}\!+\!\mathrm {FP}\!+\!\mathrm {FN}}, \mathrm {Sens}=\frac {\mathrm {TP}}{\mathrm {TP}\!+\!\mathrm {FN}}, \mathrm {Spec}=\frac {\mathrm {TN}}{\mathrm {TN}\!+\!\mathrm {FP}}. \tag {8}\end{equation*}

For sensitivity, specificity, accuracy, and F1, uncertainty was quantified using exact two-sided 95% Clopper–Pearson confidence intervals computed from one-vs-rest confusion counts aggregated across all outer-test folds. For AUROC and AUPRC, the full nested cross-validation pipeline was repeated with 10 different random seeds and 95% confidence intervals were derived from the 2.5th and 97.5th percentiles of the resulting distribution. All confidence intervals were computed at the class level using one-vs-rest evaluation.

Model interpretability was assessed at two levels. At the global level, impurity-based feature importance (Figure 6) and permutation importance (Supplementary figure S.I) were computed to provide complementary rankings of predictive features. Impurity-based feature importance was computed from the decrease in Gini impurity across all splits in the fitted Random Forest ensemble, providing a ranking of predictive features across the full cohort (Figure 6). Permutation importance was computed as a complementary measure by evaluating the decrease in out-of-bag accuracy when each feature was randomly shuffled (Supplementary figure S.I). At the instance level, the tree-ensemble structure in principle permits tracing individual predictions through the ensemble’s decision paths. While the present study focuses on global feature-importance analysis (Figure 6, Supplementary figure S.I), this architectural property enables future extension to per-patient explanations (for example via SHAP) without requiring external approximation methods, and distinguishes the chosen model family from black-box alternatives [35].

**FIGURE 4.**
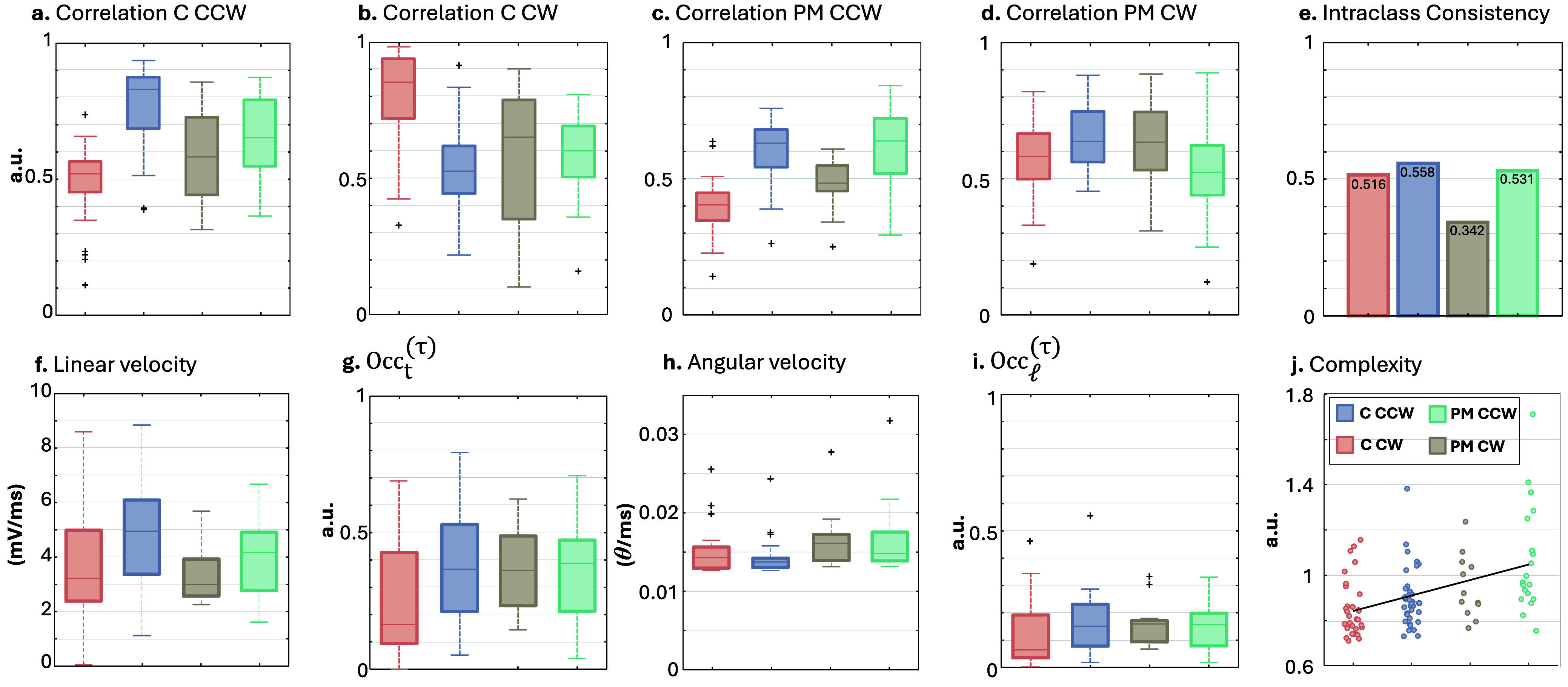
Quantitative VCG analysis across AFL subtypes. (A–D) Correlation of patient loops to archetypes (LOO within-class). (E) Intraclass consistency (PC1 variance fraction). (F) Linear speed. (G) $\mathrm {Occ}_{t}^{(\tau)}$, (H) Angular speed, (I) $\mathrm {Occ}_{\ell } ^{(\tau)}$. (J) Loop complexity. Boxes/points show fold-wise distributions. Statistical comparisons use unpaired $t$-tests with Bonferroni correction; full $p$-values in Supplementary Tables S.I-S.IV.

**FIGURE 5.**
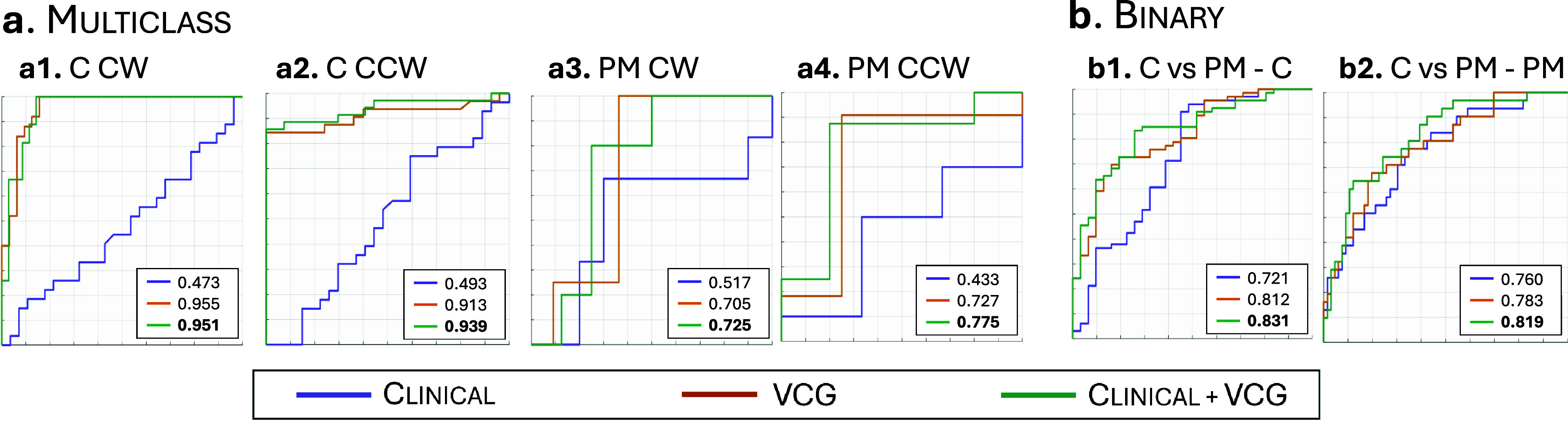
One-vs-rest AUROCs by AFL subtype for multimodal (clinical + VCG), VCG-only, and clinical-only models on the outer test folds, evaluated under a hierarchical cascade: substrate discrimination (C vs PM, Panel b) followed by conduction direction within correctly classified patients (Panel a). Multimodal models consistently yield the highest AUROCs across subtypes. Corresponding AUPRC values are reported in Supplementary Table S.VI.

**FIGURE 6.**
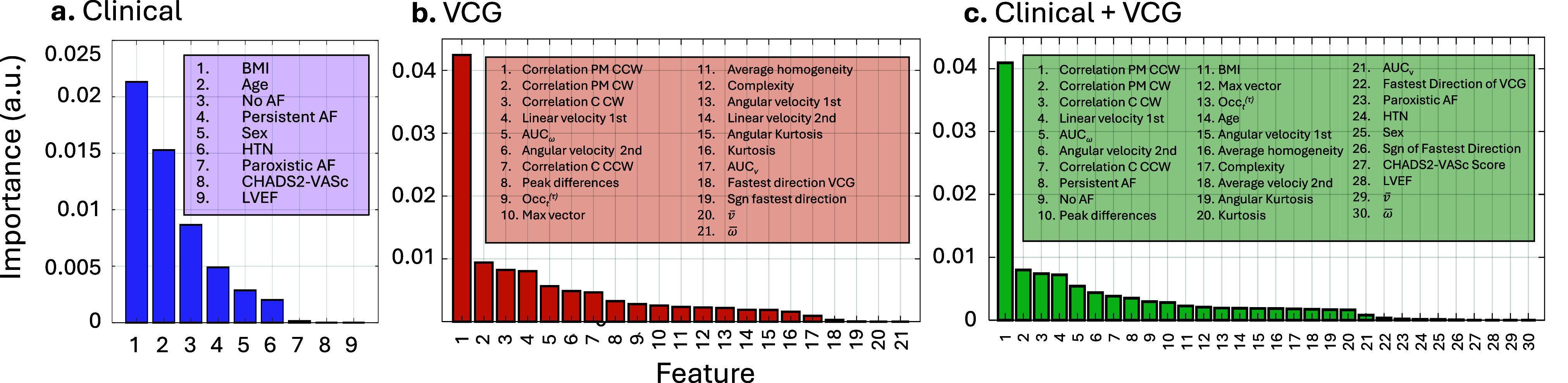
**Feature importance for Random-Forest models.**Impurity-based feature importance is shown for (a) Clinical-only model, (b) VCG-derived model, and (c) multimodal model combining clinical and VCG-derived features. Ranked importances highlight archetype-correlation and kinematic VCG descriptors (velocity, angular profile) alongside key clinical predictors (BMI, age, AF history). Permutation-based importance is provided for comparison in supplementary figure S.I. Feature definitions are in supplementary table S.X. *Abbreviations*: AF = atrial fibrillation; BMI = body-mass index; CHA_2_DS_2_-VASc = thromboembolic risk score; HTN = hypertension; LVEF = left-ventricular ejection fraction; VCG = vectorcardiogram; C = common (CTI-dependent) flutter; PM = perimitral AFL; CW/CCW = clockwise/counterclockwise; $\overline {v}=$ mean linear speed over the full atrial cycle (linear-velocity profile); $\overline {\omega }$ = mean angular speed over the full atrial cycle (angular-velocity profile); AUC${}_{v}=$ area under the linear-speed profile; AUC${}_{\omega } =$ area under the angular-velocity profile; $\mathrm {Occ}_{t}^{(\tau)}=$ temporal subthreshold occupancy at threshold $\tau =0.25$.

## IV. Results

### A. VCG–Archetype Correlation

Across the cohort, each test-set patient’s VCG loop showed the highest cosine correlation with the train-set archetype of its own AFL subtype (Table 2). Specifically, the within-class correlations were $0.832\pm 0.129$ for C_CCW_ against the C_CCW_ archetype, $0.874\pm 0.154$ for C_CW_ against the C_CW_ archetype, $0.647\pm 0.127$ for PM_CCW_ against the PM_CCW_ archetype, and $0.667\pm 0.159$ for PM_CW_ against the PM_CW_ archetype. Between-class contrasts (Bonferroni-corrected) were significant for most cross-archetype comparisons —full statistics are provided in supplementary table S.I.

**TABLE 2:** Average Correlation of Each VCG to the Four Archetypes (Test Set for the Within-Class Archetype). Values are Mean ± SD. Highest Value in Each Row is Bolded

VCG class	Arch C_CCW_	Arch C_CW_	Arch PM_CCW_	Arch PM_CW_
C_CCW_	$0.832~\pm ~0.129$	$0.565~\pm ~0.149$	$0.503~\pm ~0.102$	$0.655~\pm ~0.108$
C_CW_	$0.520~\pm ~0.128$	$0.874~\pm ~0.154$	$0.311~\pm ~0.097$	$0.593~\pm ~0.148$
PM_CCW_	$0.673~\pm ~0.157$	$0.608~\pm ~0.120$	$0.647~\pm ~0.127$	$0.564~\pm ~0.178$
PM_CW_	$0.579~\pm ~0.182$	$0.657~\pm ~0.252$	$0.411~\pm ~0.101$	$0.667~\pm ~0.159$

### B. Intraclass Consistency, Loop Complexity, and Maximal Vector

Intraclass morphological consistency (fraction of variance explained by PC1) was higher for C_CCW_ (0.560), C_CW_ (0.516) and PM_CCW_ (0.531), whereas notably lower for PM_CW_ (0.342). Group-wise differences versus PM_CW_ were significant after Bonferroni correction ($p< 0.001$); pairwise p-values for loop complexity are provided in Supplementary Table S.III. Mean loop complexity (perimeter ratio; higher denotes greater irregularity) was lowest in C_CW_ ($0.854\pm 0.127$) and C_CCW_ ($0.900\pm 0.131$), and higher in PM AFL, especially PM_CCW_ ($1.066\pm 0.240$). The maximum dipole magnitude (Max Vector) tended to be larger in common flutters (e.g., C${}_{\text {CCW}} 598.806\pm 480.617$) than in PM circuits (Table 3).

**TABLE 3:** Intraclass Consistency, Loop Complexity, and Maximum Dipole Magnitude (Max Vector) by AFL Subtype

AFL subtype	Consistency (C_1_)	Complexity	Max Vector
C_CW_	0.516	$0.854~\pm ~0.127$	$382.887~\pm ~237.901$
C_CCW_	0.560	$0.900~\pm ~0.131$	$598.806~\pm ~480.617$
PM_CW_	0.342	$0.949~\pm ~0.140$	$362.499~\pm ~100.751$
PM_CCW_	0.531	$1.066~\pm ~0.240$	$508.026~\pm ~188.430$

### C. Velocity–Profile Analysis

Vectorcardiographic linear and angular velocities, along with slow-percentage occupancy descriptors ($\mathrm {Occ}_{t}^{(\tau)}$, $\mathrm {Occ}_{\ell } ^{(\tau)}$) and their ratio ($R^{(\tau)}$), demonstrated class-dependent profiles (Table 4). Linear speed was highest in C_CCW_ ($5.276\pm 3.522$) and lower in PM circuits; angular velocity was similar across classes with slightly higher averages in PM forms. Pairwise $p$-values for linear speed indicated that C_CCW_ differed from PM_CCW_ ($p=0.0212$) and from C_CW_ ($p=0.047$). For angular velocity, C_CCW_ differed from C_CW_ ($p=0.007$) and from PM_CCW_ ($p=0.0358$). Detailed pairwise p-values for all velocity-related descriptors are in Supplementary Table S.IV, and higher-order velocity-profile descriptors (asymmetry, kurtosis, area) that complement the mean and variance summaries are provided in Supplementary Table S.II.

**TABLE 4:** Linear and Angular Velocity Metrics and Slow-Occupancy Indices by AFL Subtype (Mean ± SD)

AFL subtype	Velocity metrics		Slow-occupancy indices		
	Linear Vel.	Angular Vel.	$\mathbf {Occ}_{\ell }^{(\tau)}$	$\mathbf {Occ}_{t}^{(\tau)}$	$\mathbf {R}^{(\tau)}$
C_CW_	$3.646~\pm ~1.935$	$0.014~\pm ~0.003$	$0.062~\pm ~0.118$	$0.164~\pm ~0.194$	$2.579~\pm ~0.786$
C_CCW_	$5.276~\pm ~3.522$	$0.014~\pm ~0.002$	$0.151~\pm ~0.103$	$0.366~\pm ~0.190$	$2.389~\pm ~0.498$
PM_CW_	$3.278~\pm ~0.993$	$0.016~\pm ~0.004$	$0.158~\pm ~0.084$	$0.362~\pm ~0.164$	$2.257~\pm ~0.482$
PM_CCW_	$4.031~\pm ~1.426$	$0.015~\pm ~0.004$	$0.157~\pm ~0.082$	$0.387~\pm ~0.188$	$2.407~\pm ~0.264$

### D. Multimodal Classification Performance

We compared three models trained in a nested cross-validation setting (Figure 3): (i) a multimodal model combining VCG-derived features with clinical variables; (ii) a model using VCG-derived features only; and (iii) a model using clinical variables only. The selected ensemble used a Random Forest as the best-performing learner (see supplementary table S.IX). The classification was structured hierarchically: the first stage discriminated anatomical substrate (common vs. PM), and the second stage resolved conduction direction (CW vs. CCW) within each substrate. On held-out test folds, the multimodal model achieved strong substrate-level discrimination (Table 5, Panel A), with AUROC^1^ of 0.842 for common and 0.839 for PM subtypes. Within-substrate conduction direction was accurately resolved for common flutter (Panel B; AUROC > 0.90), while PM rotation remained more challenging, consistent with the morphological heterogeneity of this group. (table 5; Figure 5). At the substrate level, the Random Forest correctly identified 63 out of 66 common (C) patients and 14 out of 31 perimitral (PM) patients. Within-substrate conduction direction discrimination was subsequently evaluated on the 77 patients correctly classified at the substrate level (63 C and 14 PM), whose results are reported in Table 6 (see Tables 5 and S.IX for detailed metrics).Gains were most marked for common flutters (C_CCW_, C_CW_), while PM subtypes, particularly PM_CW_, remained challenging with lower sensitivity but high specificity (e.g., PM_CW_ specificity $0.988\pm 0.024$). AUPRC, which better reflects performance under class imbalance, confirmed strong substrate-level discrimination for common AFL (0.900) while highlighting the challenge of PM detection (0.667); within-substrate AUPRC further separated well-resolved common rotation (C CCW: 0.918, C CW: 0.847) from the harder PM rotation task (PM CCW: 0.743, PM CW: 0.467) (supplementary table S.VI). Internal-validation metrics from the nested CV inner loop — included to contextualize generalization and assess potential optimism in the outer-test estimates — are reported in Supplementary Table S.V. Additional test-set metrics (Accuracy, AUPRC) appear in Supplementary Table S.VI. The Random Forest was selected over AdaBoost, Decision Tree, Elastic Net, and Linear SVM based on the comparison provided in Supplementary table S.IX, which reports both internal-validation and external-test performance for all candidate models.^1^Note that, in Table 5, Sens, Spec, and F1 are reported as exact 95% Clopper–Pearson confidence intervals computed over the aggregated outer-loop test folds of the nested cross-validation scheme, while AUROC, AUPRC, and Acc are reported as median [95% CI percentile] over 10 repeated seeds (Figure 3). Both approaches replace the per-fold standard deviation previously used, providing more robust uncertainty estimates.

**TABLE 5:** Hierarchical Classification Results on the Outer Test Folds. Values are Reported as Estimate [95% CI]. Sens, Spec, and F1 Use Exact Clopper-Pearson Intervals; AUROC Uses Median [2.5th-97.5th Percentile] Over 10 Repeated Nested CV Runs, for Both Panel A (Substrate-Level) and Panel B (Within-Substrate). Additional Test-Set Metrics (Accuracy, AUPRC) are Reported in Supplementary Table S.VI

Metric	Model	A. Substrate-level discrimination			
		C subtypes		PM subtypes	
Sens	Multi	0.95 [0.87, 0.99]		0.45 [0.27, 0.64]	
	VCG	0.86 [0.76, 0.94]		0.39 [0.22, 0.58]	
	Clinical	0.83 [0.72, 0.91]		0.45 [0.27, 0.64]	
Spec	Multi	0.45 [0.27, 0.64]		0.95 [0.87, 0.99]	
	VCG	0.39 [0.22, 0.58]		0.86 [0.76, 0.94]	
	Clinical	0.45 [0.27, 0.64]		0.83 [0.72, 0.91]	
F1	Multi	0.86 [0.80, 0.91]		0.58 [0.43, 0.72]	
	VCG	0.80 [0.73, 0.86]		0.46 [0.32, 0.61]	
	Clinical	0.80 [0.72, 0.86]		0.50 [0.36, 0.64]	
AUROC	Multi	0.84 [0.81, 0.88]		0.84 [0.80, 0.87]	
	VCG	0.82 [0.78, 0.86]		0.81 [0.74, 0.84]	
	Clinical	0.72 [0.66, 0.77]		0.75 [0.70, 0.79]	
		B. Within-substrate conduction discrimination			
		C_CCW_	C_CW_	PM_CCW_	PM_CW_
Sens	Multi	0.86 [0.70, 0.95]	1.00 [0.88, 1.00]	0.89 [0.52, 1.00]	0.40 [0.05, 0.85]
	VCG	0.87 [0.70, 0.96]	1.00 [0.87, 1.00]	0.67 [0.30, 0.93]	0.33 [0.01, 0.91]
	Clinical	0.54 [0.34, 0.72]	0.56 [0.35, 0.75]	0.75 [0.35, 0.97]	0.17 [0.02, 0.64]
Spec	Multi	1.00 [0.88, 1.00]	0.86 [0.70, 0.95]	0.40 [0.05, 0.85]	0.89 [0.52, 1.00]
	VCG	1.00 [0.87, 1.00]	0.87 [0.70, 0.96]	0.33 [0.01, 0.91]	0.67 [0.30, 0.93]
	Clinical	0.56 [0.35, 0.75]	0.54 [0.34, 0.72]	0.17 [0.00, 0.64]	0.75 [0.35, 0.97]
F1	Multi	0.92 [0.83, 0.97]	0.92 [0.82, 0.97]	0.80 [0.56, 0.94]	0.50 [0.16, 0.84]
	VCG	0.93 [0.83, 0.98]	0.93 [0.83, 0.98]	0.71 [0.44, 0.90]	0.29 [0.04, 0.71]
	Clinical	0.55 [0.41, 0.68]	0.55 [0.41, 0.68]	0.63 [0.38, 0.84]	0.22 [0.03, 0.60]
AUROC	Multi	0.90 [0.89, 0.94]	0.92 [0.91, 0.95]	0.70 [0.50, 0.89]	0.76 [0.57, 0.89]
	VCG	0.91 [0.90, 0.92]	0.93 [0.91, 0.95]	0.63 [0.50, 0.90]	0.72 [0.39, 0.93]
	Clinical	0.49 [0.41, 0.57]	0.47 [0.39, 0.58]	0.42 [0.32, 0.65]	0.48 [0.20, 0.61]

**TABLE 6:** Confusion Matrix (Outer Test, Random Forest) After Hierarchical Cascade Filter. Substrate-Level Results (C vs PM): CM${}_{2\times 2}$ Reported in Text. The $4\times 4$ Matrix Reflects Only Patients Whose Substrate Was Correctly Predicted at the First Cascade Level; Off-Diagonal Zeros Between C and PM Subtypes are Structural

True / Pred.	C_CCW_	C_CW_	PM_CCW_	PM_CW_
C_CCW_	30	5	0	0
C_CW_	0	28	0	0
PM_CCW_	0	0	8	1
PM_CW_	0	0	3	2

### E. Model Explainability

For the multimodal Random-Forest classifier, impurity-based importance (Figure 6) and permutation-based importance (supplementary figure S.I) attributed predictive weight to both VCG-derived and clinical variables. Among clinical features, body-mass index, age, and history of AF ranked highly. Among VCG descriptors, correlation to archetypes, linear-velocity features across slow-velocity “blocks”, and angular-velocity descriptors were prominent contributors.

### F. Key Findings

From the extensive analyses, which are discuss in depth below, we highlight the following takeaways: (1) VCG loops align most strongly with their own AFL archetype, supporting the mechanistic specificity of the VCG representation; (2) PM CW AFL exhibits lower intraclass consistency and higher morphological complexity than other classes; (3) a hierarchical multimodal framework that fuses clinical context with VCG descriptors significantly improves substrate-level discrimination over either modality alone, and accurately resolves conduction direction for common flutter; PM rotation remains harder to identify sensitively, albeit with high specificity.

## V. Discussion

This study shows that an atrial vectorial analysis from standard 12-lead ECG enriched with clinical information can support personalized, mechanism-oriented classification of AFL subtypes. Three main observations emerge. First, atrial VCG loops display subtype-specific archetypes with high within-class similarity and significant contrasts against other classes, as quantified in the VCG-archetype correlation analysis (table 2, supplementary table S.I, and Figure 4.a-c), indicating that the atrial VCG encodes stable, mechanism-related patterns rather than idiosyncratic noise [14]. Second, geometric and kinematic descriptors differ systematically between common and PM flutters and between directions of rotation (table 3, table 4, supplementary table S.II, supplementary table S.IV,supplementary table S.II, and Figure 4.e-j). Third, when these descriptors are fused with clinical variables in an interpretable ML model, discrimination improves clearly beyond what is attainable with clinical information alone (table 5 and Figure 5).

The archetype-correlation analysis confirmed that each patient’s loop correlated most strongly with the archetype of its own AFL subtype (table 2, Figure 4.a-d), supporting the notion of “VCG fingerprints” for different macroreentrant circuits. Intraclass consistency was higher in common flutters than in PM forms, and loop complexity was lowest for common CTI-dependent and highest for PM circuits, particularly PM_CCW_ (table 3, supplementary table S.III, and Figure 4.e,j). This pattern fits with the more stereotyped anatomy of right-atrial CTI-dependent reentry [5], [6] compared with the longer, structurally heterogeneous pathways of PM circuits, often shaped by prior AF ablation or large scar areas [19], [20]. The PM_CW_ subtype combined relatively high complexity with low intraclass consistency and was indeed the most difficult class to classify, consistent with its recognized clinical heterogeneity [4], [5], [14].

Kinematic descriptors provided complementary insight into putative conduction properties. Using the linear-speed definition from the velocity-profile analysis (table 4), the non-invasively derived linear speed was highest for C_CCW_ and lower in PM circuits, and significantly helped to distinguish C_CCW_ from C_CW_, PM_CCW_ and PM_CW_ (Supplementary table A.IV and Figure 4.f, g). Angular velocity, sensitive to turning behavior around obstacles, better separated C_CCW_ from PM_CCW_ and PM_CW_ (supplementary table S.IV, Figure 4.h). Slow-occupancy indices ($\mathrm {Occ}_{t}^{(\tau)}$, $\mathrm {Occ}_{\ell } $, and $R^{(\tau)}$) consistently highlighted low-velocity regions in all subtypes (Figure 4.g,i), reinforcing the central role of slow conduction for sustaining macroreentry [3], [14]. On their own, none of these metrics fully resolves all classes, but together they provide mechanistically meaningful axes along which common and PM circuits diverge.

However, a methodological limitation of the archetype-based approach is that global loop templates may lack sensitivity to patient-specific entrance and exit zones, particularly in PM circuits where slow-conduction regions vary in location depending on prior ablation lesions and scar distribution. This is reflected in the low intraclass consistency and archetype correlation observed for PM CW. Kinematic descriptors partially mitigate this by capturing local conduction behavior independently of global morphology, but they remain summary statistics that do not explicitly encode the spatial location of slow-conduction zones along the loop. Future approaches could address this by segmenting the velocity profile into anatomically informed regions, incorporating sub-archetype clustering within PM classes, or integrating spatially resolved features derived from higher-dimensional representations of the loop trajectory.

Despite these limitations, the added value of the approach lies precisely in combining these complementary “lenses” into a single multimodal framework. The proposed model does not treat AFL subtypes as abstract labels; it integrates (i) morphology of the average atrial VCG loop (archetype similarity, orientation, complexity; Figure 4), (ii) non-invasive proxies of tissue behavior encoded in velocity and slow-conduction descriptors (table 4, supplementary table S.IV, supplementary table S.II), and (iii) demographic and clinical context such as age, body-mass index, and AF history (table 1). By learning relationships across these heterogeneous features, the Random-Forest ensemble adapts decision boundaries to each patient, effectively personalizing the predicted mechanism rather than applying fixed ECG rules. In line with this, the VCG-only model already outperformed a clinical-only model for most classes (table 5, Figure 5), but still performed poorly for PM_CW_; once clinical variables were added, the multimodal model improved sensitivity and F1-score for common flutters, maintained very high specificity (almost perfect for PM_CW_), and increased AUROC even for this challenging group (table 5, table 6, Figure 5). The added value of clinical variables was most evident for PM subtypes, where VCG morphology alone is insufficiently discriminative: within-substrate PM CCW sensitivity increased from 0.67 (VCG-only) to 0.89 (multimodal), and PM CW F1 from 0.29 to 0.50 (table 5, Panel B). For common flutter, where VCG archetypes are already highly distinctive, the marginal gain from clinical variables was expectedly smaller. The multimodal framework is therefore most beneficial precisely where discrimination is hardest and clinical context, such as prior persistent AF or left-atrial ablation history, provides information that loop morphology cannot capture. To corroborate the robustness of these findings, the Random Forest was further benchmarked against strongly regularized linear models (Elastic Net, linear SVM) to rule out overfitting in this small-sample setting. While linear models achieved competitive internal-validation performance for common subtypes, they generalized less well on held-out test folds, particularly for PM AFL, where sensitivity dropped markedly (Supplementary table S.IX). This suggests that the Random Forest captures clinically relevant non-linear interactions (such as archetype correlation conditional on AF history) that linear decision boundaries cannot represent, rather than fitting noise. The choice of tree-based ensembles over deep learning architectures was deliberate: while neural networks can achieve strong performance on larger datasets, they require post-hoc approximation methods (e.g., SHAP, GradCAM) whose attributions are not guaranteed to be faithful to the model’s actual reasoning [35]. The Random Forest’s feature-importance rankings arise from its internal split structure, offering direct (though not bias-free) insight into which features drive predictions. While impurity-based importance can overweight continuous or correlated features, the consistency with permutation-based rankings (Supplementary figure S.I) provides reassurance that the identified feature hierarchy is not an artifact of these known biases.

Beyond model selection, the hierarchical formulation further aligns the model with clinical decision-making: substrate identification (CTI-dependent vs. PM) determines lesion strategy and procedural complexity, whereas rotation direction, although informative, carries lower procedural weight. By resolving substrate first, the framework prioritizes the distinction of greatest clinical consequence.

Having established the rationale for the hierarchical multimodal framework, we now contextualize its performance within the current state of the art. The problem of discriminating AFL subtypes from the surface ECG has been addressed through several complementary avenues, yet no prior study has tackled the full four-class problem (common CCW, common CW, PM CCW, PM CW) with a multimodal, interpretable pipeline validated through nested cross-validation. Early body-surface mapping work by SippensGroenewegen et al. [7] provided detailed characterization of CCW versus CW typical flutter activation patterns but required 62-lead recordings unavailable in routine practice. Classical ECG morphology criteria, as reviewed by CosÍo [36] and Bagliani et al. [11], remain the clinical standard but suffer from limited sensitivity for atypical circuits and substantial interobserver variability, with diagnostic accuracy ranging between 50% and 80% depending on expertise [12]. W. Hu et al. [37] demonstrated that peri-mitral flutter wave morphology on the surface ECG is predominantly shaped by right atrial activation rather than by the left-atrial circuit itself, offering a mechanistic explanation for the poor discriminative power of conventional ECG criteria for PM subtypes and supporting the need for quantitative approaches such as the one proposed here. On the computational side, Luongo et al. [38] trained a hybrid in silico–clinical decision tree to discriminate three AFL location classes (CTI-dependent, peri-mitral, and other left-atrial), achieving 76.3% accuracy on a clinical test set of 38 patients with per-class sensitivities of 89.7% (CTI), 75.0% (peri-mitral), and 64.1% (other LA). While their approach benefits from synthetic data augmentation via bi-atrial statistical shape models [39], it does not resolve rotational direction within each substrate and relies on a single hold-out split rather than nested cross-validation. In a related computational framework, Luongo et al. [40] applied recurrence quantification analysis to simulated 12-lead ECGs, achieving strong in silico discrimination of up to 20 AFL mechanisms though clinical validation was limited to a single proof-of-concept patient case. Vila et al. [41] proposed directed network mapping for AFL mechanism detection, evaluating performance on simulated scenarios and a limited retrospective cohort of only 10 clinical cases. Azman et al. [15] analyzed VCG loop variability in a clinical cohort and developed formal classification models to demonstrate differences between right- and left-atrial circuits, but without a multimodal integration, or nested evaluation. Gul et al. [42] used P-to-P interval variability to distinguish focal from macroreentrant AFL mechanisms in 46 patients, but this addresses a different classification task (mechanism type rather than circuit location and rotation) and faced extreme class imbalance (41:5). Meste et al. [43] proposed SVD-based F-wave resynchronization for left versus right AFL localization in 56 patients, achieving accuracy of approximately 0.80 with simple features but without resolving rotational direction or incorporating clinical covariates. Abdala-Lizarraga et al. [44] recently reviewed advanced non-invasive techniques for CTI-dependent flutter characterization, including VCG and body-surface potential mapping, and noted the promising but still preliminary nature of these approaches for guiding ablation planning. Our study advances these prior efforts in several key respects. First, we address a finer-grained four-class problem that directly maps onto distinct ablation strategies, whereas most prior studies address two- or three-class discrimination [7], [15], [38], [43]. Second, we demonstrate that fusing VCG-derived mechanistic descriptors with clinical variables improves AUROC from 0.63–0.93 (VCG-only) to 0.70–0.92 (multimodal) across subtypes, with the largest gains for PM classes where clinical context (prior persistent AF, ablation history) provides information inaccessible to signal-based methods alone. The substrate-level AUROC of 0.84 and within-substrate common flutter AUROC exceeding 0.90 compare favorably with the 76.3% accuracy reported by Luongo et al. [38] for a similar but coarser discrimination task on a comparable clinical sample size, while additionally resolving conduction direction. Third, we employ nested cross-validation with 10 repeated seeds and report Clopper–Pearson confidence intervals and percentile-based AUROC intervals, providing more robust uncertainty estimates than single hold-out splits [45]. Fourth, unlike deep learning approaches that treat the ECG as a black-box input [22]—limitation highlighted by Pantelidis et al. [21], our pipeline produces interpretable, mechanistically grounded features (archetype correlation, slow-velocity occupancy, geometric complexity) whose clinical relevance can be directly assessed by electrophysiologists [35]. Nevertheless, direct comparison across studies is limited by differences in class definitions, patient populations, ground-truth procedures, and evaluation protocols, and the modest PM sample sizes in the present cohort result in wide confidence intervals for these subtypes (Table 5). Standardized benchmarks and prospective multicenter validation remain essential to establish the relative merits of these approaches [46].

Complementing this comparative perspective, interpretability and reproducibility are essential for clinical uptake. The pipeline is modular and FAIR-aligned [32], with explicit steps for ECG preprocessing, atrial-segment selection, VCG reconstruction, feature extraction, and model training (Figure 2), which facilitates external validation and extensions. Feature definitions corresponding to the importance rankings in Figure 6 are provided in Supplementary table S.X to facilitate interpretation of the multimodal model’s explanatory outputs. Accordingly, feature-importance analysis (Figure 6, supplementary table S.X) showed that, in the clinical-only model (table 6.a), body-mass index, age, and especially persistent AF history are the most influential variables, consistent with the known association between structural remodeling, AF burden, and complex left-atrial circuits [19], [20]. In the multimodal setting (Figure 6.c), archetype correlations and kinematic descriptors dominate the top ranks, with persistent AF and body-mass index still featuring among the most relevant clinical covariates. The signal-only model (Figure 6.b) shows a similar prominence of correlation and velocity-profile metrics. This agrees with prior observations that VCG loops exhibit zones of point-density accumulation consistent with slow-conduction regions [14] and suggests that the model is leveraging both anatomic–functional information and patient-level risk factors. Because tree-based ensembles readily support per-patient explanations (e.g., via local feature-attribution methods), the same framework could, in a prospective setting, provide not only a predicted AFL subtype but also a concise rationale tailored to each individual. We note that the interpretability analysis presented here operates at the global level (identifying which features are most influential across the cohort) rather than providing per-patient explanations of individual predictions. The latter is architecturally supported by the tree-ensemble structure and will be implemented via SHAP in the planned prospective validation, where per-patient rationales can be generated and evaluated by electrophysiologists for clinical face validity. Feature importance was assessed via impurity-based and permutation methods. While there was consistency between impurity-based rankings (Figure 6) and permutation importance (Supplementary figure S.I), impurity-based importance is known to be biased toward high-cardinality continuous features and to dilute importance across correlated predictors, so the reported rankings should be interpreted as indicative rather than definitive. Model-agnostic approaches such as SHAP would provide complementary, less bias-prone instance-level explanations and will be incorporated in the planned external validation and prospective multicenter studies to further strengthen per-patient clinical interpretability.

On a practical level, a prerequisite for this methodology is the availability of an atrial signal that is minimally contaminated by ventricular activity. In AFL, a 2:1 AV conduction ratio is frequent, so P waves are often masked by QRS or T waves [47]. In our dataset, adenosine-induced AV block was used to obtain short intervals with clear atrial activity, but this is not always feasible or desirable in routine practice. Because atrial loops are highly consistent within subtypes, even a single clean cycle may be sufficient if longer RR intervals are available. Nevertheless, improved algorithms to retrieve the atrial signal directly from the ECG would be valuable. Classical QRS–T cancellation approaches [13], [48] risk removing atrial activity when cycles are tightly coupled to ventricular events, and blind source separation methods [49] typically yield only one atrial-dominated component—insufficient to reconstruct a full 3D VCG, for which at least two or three components are required. More advanced methods include exploiting spatial or temporal structure, such as projective filtering [50], periodic component analysis ($\pi $CA) [51], robust wavelets [52], or phase or transforms with clinical rules for optimizing P wave detection [53], [54]. These and recent T-wave–removal strategies proposed [55] could, if robustly extended to AFL, allow reconstruction of complete atrial cycles even under stable 2:1 conduction.

Several limitations should be acknowledged. This is a retrospective, single-center study with a moderate sample size and class imbalance; PM AFL, especially PM_CW_, are underrepresented, which likely contributes to lower sensitivity and wider uncertainty for this class, although the cohort improves on prior VCG studies in terms of numbers and subtype coverage [14]. No resampling or class-weighting strategies were applied, as synthetic augmentation of the smallest subgroups (PM CW, n = 12) risks introducing artificial feature patterns in a high-dimensional, multimodal feature space; future work on larger, prospectively collected cohorts (including targeted enrichment of PM subtypes) will enable evaluation of these techniques and more robust estimation of PM-specific performance. As expected, uncertainty was widest for the PM subtypes, particularly PM CW, reflecting the limited number of available cases. Exact Clopper-Pearson intervals for threshold-based metrics and repeated-nested-CV intervals for AUROC and AUPRC provided a more stable and transparent characterization of uncertainty than fold-wise standard deviations alone. For PM CW, interval width remained substantial across metrics (e.g., sensitivity 0.40 [0.05, 0.85]; AUROC 0.764 [0.567, 0.891]), indicating that performance estimates for this subgroup should be interpreted cautiously pending validation in larger cohorts. Additionally, only common CTI-dependent and PM AFL were analyzed; other macroreentrant circuits (e.g., upper- or lower-loop right-atrial reentry, roof-dependent left-atrial flutter) were not represented and will require specific archetypes and possibly additional features. Several echocardiographic markers of atrial remodeling—including indexed left atrial volume, right atrial area, E/e’ ratio, and valvular regurgitation severity—are reported descriptively in table 1 but were not included as predictive features due to 16–28% missingness in this retrospective cohort, as echocardiography was not part of the standardized EP protocol and is inconsistently reported even across Level 1 registry standards [46]. Future prospective studies should systematically collect volumetric and diastolic markers to evaluate their incremental predictive value and to disentangle the relative contributions of atrial remodeling and VCG morphology to AFL subtype discrimination. Beyond cohort-specific constraints, external validation in independent cohorts with different demographics and acquisition systems is needed to fully establish generalizability, beyond the robust but inherently internal estimates provided by nested cross-validation (Figure 3, supplementary table S.V, supplementary table S.VI, supplementary table S.IX). The arithmetic mean used to construct subtype archetypes may be sensitive to outlier loops, particularly in small or heterogeneous classes such as PM CW; robust alternatives (e.g., medoid, component-wise median, Fréchet mean) each carry their own trade-offs and, together with sub-archetype discovery, warrant exploration in larger cohorts. The slow-velocity threshold ($\tau = 0.25$) was fixed a priori based on empirical observations [14]; a systematic sensitivity analysis over a range of thresholds could refine the optimal operating point and is warranted in larger cohorts. From a modeling perspective, the VCG descriptors adopt a geometrical, data-driven viewpoint and do not yet arise from explicit biophysical models of atrial activation. The geometric complexity metric relies on a 2D PCA projection; while the third principal component was minor (< 10%) for most subtypes, it reached $\approx 13\%$ for PM CW (Supplementary table S.VIII), suggesting that a 3D extension may better characterize left-atrial circuits with complex out-of-plane geometry. The IDT assumes a fixed torso geometry that may not fully capture inter-patient variability due to atrial remodeling, enlargement, or electrode placement differences [31]; future work should compare alternative transforms—such as the Kors regression method [27], P-wave–optimized matrices [29], and data-driven approaches [24] —specifically for atrial flutter signals.

Looking ahead, in-silico 3D atrial models incorporating realistic ionic mechanisms (e.g., Courtemanche-type formulations [56], [57], [58]) and forward computation of body-surface potentials could generate synthetic VCG loops for different macroreentrant circuits, which in turn could be compared with archetypes derived from real data to refine mechanistic interpretation [38]. On the invasive side, we are constrained by the spatial resolution and coverage of navigation systems; yet, not all cases were recorded with the highest density catheters such as Advisor^TM^ HD Grid Mapping Catheter (Abbott Laboratories) [59] and other high-density catheters (e.g. OPTRELL^TM^ Catheter [60] and RHYTHMIA HDx^TM^ [61]) and emerging mapping systems [62], [63] may acquire more detailed activation maps and provide richer and other versions of ground truth for correlating invasive patterns with non-invasive VCG features. Future work combining high-density mapping, in-silico modeling, advanced atrial-signal extraction, and the kind of multimodal VCG–clinical analysis proposed here could lead to a more comprehensive, mechanism-based, and patient-specific framework for non-invasive AFL subtype identification and subsequent management.

## VI. Conclusion

We presented a multimodal, patient-specific framework for non-invasive AFL mechanism identification that fuses atrial VCG-derived morphological and kinematic descriptors with clinical variables in an interpretable, hierarchical Random-Forest classifier. The pipeline produces mechanistically grounded, per-patient estimates of the underlying macroreentrant circuit before invasive mapping, with the potential to streamline ablation planning and focus mapping efforts on the most likely substrate. The framework is FAIR-aligned and designed for reproducible deployment across centers. Prospective, multicenter studies—ideally combined with advanced atrial-signal extraction methods, high-density mapping, in-silico modeling, and instance-level explainability via SHAP—will be crucial to validate and extend these findings and to bring non-invasive mechanism-based AFL stratification closer to routine clinical practice.

## Conflicts of Interest

SRC reports equity, intellectual property, and consulting with Physcade Inc. JMe reports fees and honoraria for lectures, education and scientific advice from Abbott, Biotronik, Daiichi-Sankyo, Everpace, iRhythm, Johnson&Johnson & Zoll. These are not related to this work. The rest of authors declare no known interests.

## Ethical Statement

Data were collected under clinical care at Hospital Universitario La Paz (Madrid, Spain). All data were extracted from an institutional EP database and analyzed retrospectively with approval from the Institutional Review Board. No identifiable patient information was used. Written informed consent was obtained in accordance with the Declaration of Helsinki and applicable regulations.

## Supplementary Materials

Supplementary Materials
