# Supplementary material for: Multimodal Patient-Specific Identification of Atrial Flutter Circuits From ECG Time Series Using Explainable Machine Learning
Source: IEEE J Transl Eng Health Med. 2026 May 19;14:248–62. doi: 10.1109/JTEHM.2026.3694616 (PMC13278741; doi:10.1109/JTEHM.2026.3694616)
Supplement: Supplementary Materials [file jtehm-3694616-mm.zip › jtehm-3694616-mm/JTEHM3694616_appendices.pdf]

Multimodal Patient-Specific Identification of Atrial Flutter Circuits from ECG Time Series Using Explainable Machine Learning

Samuel Ruipérez-Campillo[1,*], David Hernando[2], Elisa Ramírez[2], Sergio Castrejón[3], Cecilia Zapata[3], Carlos Rodríguez Carneiro[5], Julia E. Vogt[1], José Luis Merino[3], Francisco Castells[2], José Millet[2]

APPENDICES: SUPPLEMENTARY TABLES AND ADDITIONAL RESULTS

The supplementary material provides detailed statistical outputs and modeling details that support the main Results section. Section A report supplementary statistics VCG-derived analyses. Section B provide complementary classification metrics from the nested cross-validation framework, including inner-loop validation performance and model-family comparisons. Lastly, section C document the implementation details for Random-Forest hyperparameter selections across outer folds and define the predictors used in the feature-importance analysis.

APPENDIX A
SUPPLEMENTARY STATISTICAL ANALYSES

Supplementary table S.I reports Bonferroni-corrected pairwise p-values for differences in cosine correlations between patient VCG loops and the subtype archetypes. These comparisons complement the summary correlations presented in the main text and quantify which cross-archetype contrasts are statistically supported.

TABLE S.I: P-values (Bonferroni-corrected) for between-group comparisons of VCG–archetype correlations.

	C_{CW} Arch	C_{CCW} Arch	PM_{CW} Arch	PM_{CCW} Arch
C_{CW}	–	< 0.001	1	1
C_{CCW}	< 0.001	–	1	1
PM_{CW}	< 0.001	< 0.001	–	0.385
PM_{CCW}	< 0.001	< 0.001	0.009	–

Supplementary table S.III provides Bonferroni-corrected pairwise p-values for the loop-complexity metric across AFL subtypes (upper triangle). This table supports the interpretation of between-subtype differences in morphological irregularity beyond the descriptive statistics reported in the main Results.

Supplementary table S.IV reports Bonferroni-corrected pairwise p-values for velocity-related descriptors, including linear velocity, angular velocity, and slow-percentage occupancy descriptors. These tests formally quantify the subtype-specific differences referenced in the velocity-profile Results section.

Supplementary table S.II extends the velocity-profile characterization by providing higher-order descriptors (asymmetry and kurtosis) and discrete integrals (areas) of the linear and angular velocity profiles. These quantities complement mean and variance summaries by capturing profile shape, peakedness, and aggregate magnitude over the atrial cycle.

APPENDIX B
SUPPLEMENTARY CLASSIFICATION RESULTS

Supplementary Table S.V reports performance on the inner-loop validation folds of the nested cross-validation scheme (i.e., internal test sets used during hyperparameter selection), including Accuracy and AUPRC alongside the primary metrics. These results are provided to contextualize the generalization metrics reported on the outer-loop test folds in the main results, enabling direct comparison across evaluation stages.

Supplementary table S.VI provides additional outer-loop test-fold metrics (Accuracy and AUPRC) to complement the main reported sensitivity, specificity, F1-score, and AUROC.

Supplementary table S.IX compares five model families (Random Forest, AdaBoost, Decision Tree, Elastic Net, and Linear SVM) across multiple metrics. Panel A reports the internal-validation performance used for model selection; these Random Forest values correspond to those in Supplementary table S.V. Panel B reports the external outer-test-fold performance; these Random Forest values correspond to table V. This dual reporting ensures full traceability of the model selection process.

APPENDIX C
SUPPLEMENTARY IMPLEMENTATION AND DOCUMENTATION

Supplementary table S.VII lists the optimal Random-Forest hyperparameter configuration selected in each outer fold of the nested cross-validation. This table is provided for transparency and reproducibility of the reported performance estimates.

Supplementary table S.X defines the set of predictors used in the feature-importance analysis, including clinical variables and VCG-derived descriptors. These definitions correspond to the features displayed in the importance plots and facilitate interpretation of the multimodal model's explanatory outputs.

TABLE S.II: Asymmetry, kurtosis, and discrete integral (area) of linear and angular velocity profiles by AFL subtype.

	**Linear Velocity Profile**			**Angular Velocity Profile**		
**AFL type**	**Asymmetry**	**Kurtosis**	**Area**	**Asymmetry**	**Kurtosis**	**Area**
C_{CW}	0.432 ± 0.069	2.369 ± 1.377	1605.945 ± 963.837	0.296 ± 0.066	8.096 ± 8.192	7.143 ± 1.396
C_{CCW}	0.389 ± 0.064	2.763 ± 0.959	2468.096 ± 1757.281	0.320 ± 0.089	8.837 ± 34.138	6.845 ± 1.033
PM_{CW}	0.411 ± 0.054	3.223 ± 0.999	1491.465 ± 495.521	0.327 ± 0.086	6.187 ± 9.916	8.042 ± 1.980
PM_{CCW}	0.399 ± 0.050	2.699 ± 0.958	2077.667 ± 711.181	0.316 ± 0.057	7.035 ± 56.236	7.409 ± 2.171

TABLE S.III: Pairwise p-values (Bonferroni-corrected) for loop complexity across AFL subtypes (upper triangle shown).

	$\mathbf{C_{CCW}}$	$\mathbf{C_{CW}}$	$\mathbf{PM_{CCW}}$	$\mathbf{PM_{CW}}$
$\mathbf{C_{CCW}}$	–	0.306	0.013	1
$\mathbf{C_{CW}}$		–	< 0.001	0.150
$\mathbf{PM_{CCW}}$			–	0.970
$\mathbf{PM_{CW}}$				–

TABLE S.IV: Pairwise p-values (Bonferroni-corrected) for velocity-related descriptors.

	$\mathbf{C_{CCW}}$	$\mathbf{C_{CW}}$	$\mathbf{PM_{CCW}}$	$\mathbf{PM_{CW}}$
**Linear Velocity**				
$\mathbf{C_{CCW}}$	–	0.047	0.632	0.021
$\mathbf{C_{CW}}$		–	1	1
$\mathbf{PM_{CCW}}$			–	0.971
$\mathbf{PM_{CW}}$				–
**Angular Velocity**				
$\mathbf{C_{CCW}}$	–	1	0.007	0.036
$\mathbf{C_{CW}}$		–	0.643	0.409
$\mathbf{PM_{CCW}}$			–	1
$\mathbf{PM_{CW}}$				–
$\mathbf{Occ}_{\ell}^{(\tau)}$				
$\mathbf{C_{CCW}}$	–	0.388	1	1
$\mathbf{C_{CW}}$		–	0.435	0.462
$\mathbf{PM_{CCW}}$			–	1
$\mathbf{PM_{CW}}$				–
$\mathbf{Occ}_{t}^{(\tau)}$				
$\mathbf{C_{CW}}$	–	0.131	0.279	0.259
$\mathbf{C_{CCW}}$		–	1	1
$\mathbf{PM_{CCW}}$			–	1
$\mathbf{PM_{CW}}$				–
$\mathbf{R}^{(\tau)}$				
$\mathbf{C_{CW}}$	–	1	1	1
$\mathbf{C_{CCW}}$		–	1	1
$\mathbf{PM_{CCW}}$			–	1
$\mathbf{PM_{CW}}$				–

TABLE S.V: Internal-validation results (validation folds in nested cross-validation): all metrics reported as estimate [95% CI]. Sens, Spec, F1, and Acc use exact Clopper-Pearson intervals; AUROC and AUPRC use median [2.5th-97.5th percentile] over 10 repeated nested CV runs, for both Panel A (substrate-level) and Panel B (within-substrate).

		**A. Substrate-level discrimination**	
**Metric**	**Model**	**C subtypes**	**PM subtypes**
Sens	Multi	0.91 [0.87, 0.95]	0.41 [0.31, 0.52]
	VCG	0.91 [0.86, 0.95]	0.44 [0.34, 0.55]
	Clinical	0.87 [0.81, 0.91]	0.42 [0.32, 0.53]
Spec	Multi	0.41 [0.31, 0.52]	0.91 [0.87, 0.95]
	VCG	0.44 [0.34, 0.55]	0.91 [0.86, 0.95]
	Clinical	0.42 [0.32, 0.53]	0.87 [0.81, 0.91]
F1	Multi	0.83 [0.80, 0.87]	0.51 [0.43, 0.60]
	VCG	0.84 [0.80, 0.87]	0.54 [0.46, 0.62]
	Clinical	0.81 [0.77, 0.85]	0.49 [0.41, 0.57]
Acc	Multi	0.75 [0.71, 0.79]	0.75 [0.71, 0.79]
	VCG	0.74 [0.68, 0.76]	0.74 [0.68, 0.76]
	Clinical	0.73 [0.72, 0.77]	0.73 [0.72, 0.77]
AUROC	Multi	0.79 [0.77, 0.83]	0.79 [0.75, 0.81]
	VCG	0.78 [0.73, 0.81]	0.75 [0.71, 0.78]
	Clinical	0.70 [0.67, 0.74]	0.73 [0.69, 0.75]
AUPRC	Multi	0.89 [0.87, 0.91]	0.60 [0.57, 0.65]
	VCG	0.88 [0.84, 0.90]	0.56 [0.52, 0.64]
	Clinical	0.82 [0.76, 0.83]	0.57 [0.48, 0.61]

		**B. Within-substrate conduction discrimination**			
		$\mathbf{C_{CCW}}$	$\mathbf{C_{CW}}$	$\mathbf{PM_{CCW}}$	$\mathbf{PM_{CW}}$
Sens	Multi	0.84 [0.75, 0.90]	0.88 [0.79, 0.94]	0.81 [0.62, 0.94]	0.36 [0.11, 0.69]
	VCG	0.86 [0.77, 0.92]	0.89 [0.80, 0.95]	0.90 [0.73, 0.98]	0.25 [0.05, 0.57]
	Clinical	0.54 [0.44, 0.63]	0.46 [0.35, 0.56]	0.32 [0.20, 0.45]	0.17 [0.06, 0.33]
Spec	Multi	0.88 [0.79, 0.94]	0.84 [0.75, 0.90]	0.36 [0.11, 0.69]	0.81 [0.62, 0.94]
	VCG	0.89 [0.80, 0.95]	0.86 [0.77, 0.92]	0.25 [0.05, 0.57]	0.90 [0.73, 0.98]
	Clinical	0.56 [0.49, 0.64]	0.77 [0.70, 0.82]	0.87 [0.82, 0.91]	0.96 [0.93, 0.98]
F1	Multi	0.86 [0.80, 0.91]	0.85 [0.79, 0.90]	0.79 [0.66, 0.88]	0.40 [0.19, 0.64]
	VCG	0.88 [0.82, 0.92]	0.86 [0.80, 0.91]	0.81 [0.70, 0.90]	0.33 [0.13, 0.59]
	Clinical	0.47 [0.41, 0.54]	0.46 [0.39, 0.54]	0.34 [0.25, 0.44]	0.23 [0.13, 0.37]
Acc	Multi	0.88 [0.85, 0.90]	0.88 [0.85, 0.90]	0.70 [0.63, 0.81]	0.70 [0.63, 0.81]
	VCG	0.89 [0.86, 0.90]	0.89 [0.86, 0.90]	0.67 [0.53, 0.81]	0.67 [0.53, 0.81]
	Clinical	0.58 [0.51, 0.60]	0.58 [0.51, 0.60]	0.51 [0.44, 0.59]	0.51 [0.44, 0.59]
AUROC	Multi	0.91 [0.89, 0.92]	0.92 [0.90, 0.94]	0.71 [0.61, 0.83]	0.69 [0.59, 0.82]
	VCG	0.91 [0.89, 0.92]	0.93 [0.90, 0.94]	0.65 [0.56, 0.82]	0.67 [0.56, 0.79]
	Clinical	0.56 [0.53, 0.61]	0.56 [0.52, 0.58]	0.44 [0.30, 0.58]	0.43 [0.32, 0.50]
AUPRC	Multi	0.94 [0.93, 0.95]	0.87 [0.81, 0.91]	0.81 [0.68, 0.88]	0.44 [0.40, 0.60]
	VCG	0.94 [0.93, 0.95]	0.86 [0.82, 0.89]	0.80 [0.70, 0.87]	0.36 [0.30, 0.50]
	Clinical	0.55 [0.52, 0.58]	0.51 [0.50, 0.58]	0.56 [0.52, 0.63]	0.35 [0.24, 0.43]

TABLE S.VI: Additional test-set metrics: Accuracy and AUPRC by class and model, reported as estimate [95% CI]. Acc uses exact Clopper-Pearson intervals; AUPRC uses median [2.5th-97.5th percentile] over 10 repeated nested CV runs, for both Panel A (substrate-level) and Panel B (within-substrate).

A. Substrate-level discrimination

Metric	Model	C subtypes	PM subtypes
Acc	Multi	0.79 [0.74, 0.83]	0.79 [0.74, 0.83]
	VCG	0.77 [0.73, 0.80]	0.77 [0.73, 0.80]
	Clinical	0.73 [0.66, 0.76]	0.73 [0.66, 0.76]
AUPRC	Multi	0.90 [0.88, 0.93]	0.67 [0.58, 0.75]
	VCG	0.89 [0.87, 0.91]	0.61 [0.52, 0.68]
	Clinical	0.80 [0.76, 0.86]	0.55 [0.46, 0.60]

B. Within-substrate conduction discrimination

Metric	Model	C_{CCW}	C_{CW}	PM_{CCW}	PM_{CW}
Acc	Multi	0.90 [0.85, 0.92]	0.90 [0.87, 0.92]	0.64 [0.53, 0.89]	0.64 [0.53, 0.89]
	VCG	0.89 [0.87, 0.92]	0.89 [0.87, 0.92]	0.68 [0.46, 0.82]	0.68 [0.46, 0.82]
	Clinical	0.54 [0.43, 0.60]	0.54 [0.43, 0.60]	0.50 [0.36, 0.60]	0.50 [0.36, 0.60]
AUPRC	Multi	0.92 [0.91, 0.94]	0.85 [0.80, 0.88]	0.74 [0.52, 0.91]	0.47 [0.17, 0.63]
	VCG	0.92 [0.89, 0.93]	0.84 [0.80, 0.89]	0.75 [0.65, 0.90]	0.35 [0.14, 0.60]
	Clinical	0.48 [0.46, 0.55]	0.47 [0.39, 0.53]	0.54 [0.43, 0.60]	0.38 [0.23, 0.46]

TABLE S.VII: Hyperparameter search space across models and optimal Random-Forest configurations per outer fold.

(A) Hyperparameter search space across models

Model	Hyperparameter	Searched values
Random Forest	Number of trees	{50, 100, 150, 200}
	Minimum leaf size	{1, 5}
	Maximum splits	{100, 200}
Elastic Net	Regularization λ	{0.0001, 0.001, 0.01, 0.1}
Linear SVM	Margin parameter C	{0.01, 0.1, 1, 10}
AdaBoost	Number of trees	{50, 100, 150, 200}
	Minimum leaf size	{1, 5}
	Maximum splits	{100, 200}
Decision Tree	Minimum leaf size	{1, 5}
	Maximum splits	{100, 200}

(B) Optimal Random-Forest configuration per outer fold

Outer fold	Trees	Min. leaf size	Max splits
1	150	1	200
2	50	5	100
3	50	1	100
4	100	1	100

Notes: For the Linear SVM, polynomial, Gaussian, and RBF kernels were also evaluated; the linear kernel was selected as best-performing. Selected configurations per outer fold for the Random Forest are shown below.

TABLE S.VIII: Variance explained by the first three principal components of the atrial VCG loop, by AFL subtype.

Subtype	C1 (%)	C2 (%)	C3 (%)
**C CCW**	55.8	21.5	6.0
**C CW**	51.6	21.8	7.9
**PM CCW**	53.1	15.4	8.0
**PM CW**	34.2	22.5	12.9

Notes: Values are percentages computed from PCA on the matrix of representative loops per class. C1 and C2 span the projection plane used for the complexity metric (Eq. 2); C3 quantifies out-of-plane variance.

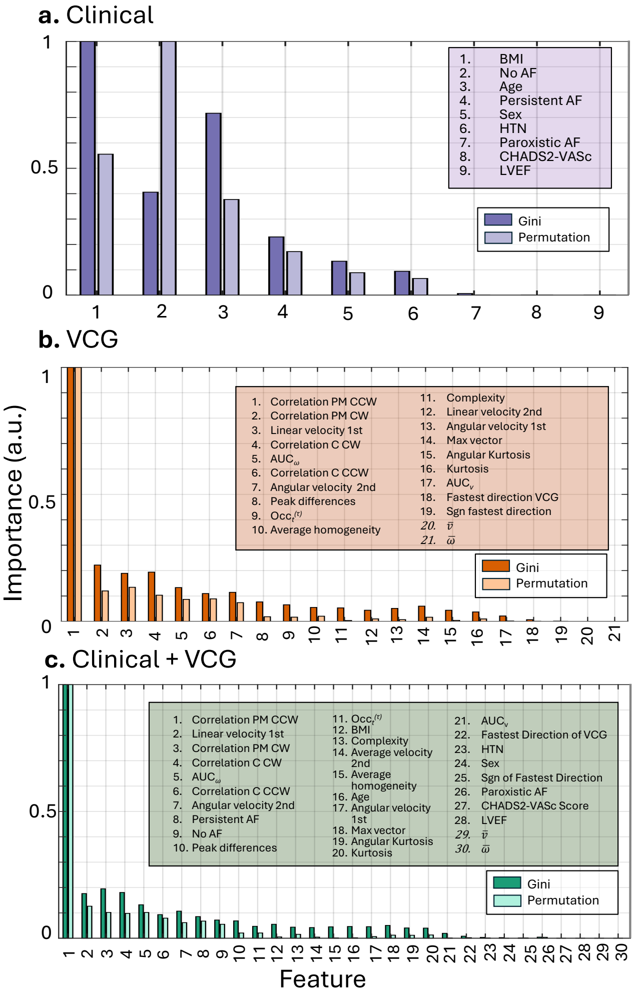

Fig. S.I: Expanding on figure 6, permutation-based and impurity-based feature importance Random-Forest models: (a) Clinical-only model, (b) VCG-derived model, and (c) multi-modal model combining clinical and VCG-derived features. See Section III for implementation details. Feature definitions are provided in Supplementary table S.X.

TABLE S.IX: Model comparison across metrics for (A) internal validation and (B) external outer-test performance using a hierarchical cascade filter. Panels A1 (top left) and B1 (bottom left) report substrate-level discrimination (C vs PM, all patients). Panels A2 (top right) and B2 (bottom right) report within-substrate discrimination (CCW vs CW, cascade filter applied). Sens, Spec, and F1: point estimate [95% CI Clopper–Pearson] (single seed). AUROC and AUPRC: median [95% CI percentile] over 10 repeated seeds.

A1. Internal Validation — Substrate (C vs PM)

		C	PM
Sens	RF	0.91 [0.87, 0.95]	0.41 [0.31, 0.52]
	Ada	0.85 [0.80, 0.90]	0.58 [0.47, 0.68]
	DT	0.76 [0.69, 0.82]	0.46 [0.36, 0.57]
	El.Net	0.88 [0.83, 0.92]	0.46 [0.36, 0.57]
	L.SVM	0.93 [0.89, 0.96]	0.31 [0.22, 0.42]
Spec	RF	0.41 [0.31, 0.52]	0.91 [0.87, 0.95]
	Ada	0.58 [0.47, 0.68]	0.85 [0.80, 0.90]
	DT	0.46 [0.36, 0.57]	0.76 [0.69, 0.82]
	El.Net	0.46 [0.36, 0.57]	0.88 [0.83, 0.92]
	L.SVM	0.31 [0.22, 0.42]	0.93 [0.89, 0.96]
F1	RF	0.83 [0.80, 0.87]	0.51 [0.43, 0.60]
	Ada	0.83 [0.79, 0.87]	0.61 [0.54, 0.69]
	DT	0.75 [0.71, 0.80]	0.47 [0.39, 0.54]
	El.Net	0.83 [0.79, 0.86]	0.54 [0.46, 0.62]
	L.SVM	0.83 [0.79, 0.86]	0.43 [0.34, 0.52]
AUROC	RF	0.79 [0.77, 0.83]	0.79 [0.75, 0.81]
	Ada	0.69 [0.68, 0.75]	0.70 [0.68, 0.74]
	DT	0.68 [0.62, 0.71]	0.67 [0.62, 0.71]
	El.Net	0.79 [0.76, 0.84]	0.77 [0.69, 0.83]
	L.SVM	0.71 [0.64, 0.76]	0.76 [0.69, 0.78]
AUPRC	RF	0.89 [0.87, 0.91]	0.60 [0.57, 0.65]
	Ada	0.81 [0.79, 0.84]	0.51 [0.48, 0.54]
	DT	0.45 [0.35, 0.58]	0.41 [0.37, 0.48]
	El.Net	0.89 [0.86, 0.92]	0.59 [0.52, 0.66]
	L.SVM	0.81 [0.77, 0.85]	0.55 [0.48, 0.59]

A2. Internal Validation — Within-substrate (cascade filter)

		C_{CCW}	C_{CW}	PM_{CCW}	PM_{CW}
Sens	RF	0.84 [0.75, 0.90]	0.88 [0.79, 0.94]	0.81 [0.62, 0.94]	0.36 [0.11, 0.69]
	Ada	0.82 [0.73, 0.90]	0.79 [0.69, 0.88]	0.83 [0.68, 0.93]	0.69 [0.39, 0.91]
	DT	0.81 [0.70, 0.89]	0.81 [0.70, 0.89]	0.76 [0.55, 0.91]	0.44 [0.22, 0.69]
	El.Net	0.93 [0.85, 0.97]	0.89 [0.80, 0.95]	0.70 [0.50, 0.86]	0.31 [0.11, 0.59]
	L.SVM	0.91 [0.83, 0.96]	0.75 [0.64, 0.83]	0.70 [0.47, 0.87]	0.50 [0.12, 0.88]
Spec	RF	0.88 [0.79, 0.94]	0.84 [0.75, 0.90]	0.36 [0.11, 0.69]	0.81 [0.62, 0.94]
	Ada	0.79 [0.69, 0.88]	0.82 [0.73, 0.90]	0.69 [0.39, 0.91]	0.83 [0.68, 0.93]
	DT	0.81 [0.70, 0.89]	0.81 [0.70, 0.89]	0.44 [0.22, 0.69]	0.76 [0.55, 0.91]
	El.Net	0.89 [0.80, 0.95]	0.93 [0.85, 0.97]	0.31 [0.11, 0.59]	0.70 [0.50, 0.86]
	L.SVM	0.75 [0.64, 0.83]	0.91 [0.83, 0.96]	0.50 [0.12, 0.88]	0.70 [0.47, 0.87]
F1	RF	0.86 [0.80, 0.91]	0.85 [0.79, 0.90]	0.79 [0.66, 0.88]	0.40 [0.19, 0.64]
	Ada	0.82 [0.76, 0.88]	0.79 [0.72, 0.86]	0.86 [0.76, 0.93]	0.62 [0.42, 0.79]
	DT	0.81 [0.74, 0.87]	0.80 [0.73, 0.86]	0.70 [0.56, 0.82]	0.50 [0.32, 0.68]
	El.Net	0.92 [0.87, 0.95]	0.90 [0.84, 0.94]	0.67 [0.53, 0.79]	0.34 [0.18, 0.54]
	L.SVM	0.85 [0.80, 0.90]	0.81 [0.74, 0.87]	0.76 [0.61, 0.88]	0.38 [0.15, 0.65]
AUROC	RF	0.91 [0.89, 0.92]	0.92 [0.90, 0.94]	0.71 [0.61, 0.83]	0.69 [0.59, 0.82]
	Ada	0.83 [0.80, 0.89]	0.88 [0.87, 0.93]	0.66 [0.58, 0.74]	0.64 [0.50, 0.76]
	DT	0.87 [0.84, 0.90]	0.86 [0.83, 0.89]	0.58 [0.47, 0.72]	0.60 [0.47, 0.65]
	El.Net	0.94 [0.92, 0.96]	0.94 [0.92, 0.96]	0.58 [0.39, 0.71]	0.58 [0.32, 0.71]
	L.SVM	0.91 [0.88, 0.94]	0.91 [0.89, 0.94]	0.43 [0.20, 0.72]	0.39 [0.21, 0.76]
AUPRC	RF	0.94 [0.93, 0.95]	0.87 [0.81, 0.91]	0.81 [0.68, 0.88]	0.44 [0.40, 0.60]
	Ada	0.88 [0.82, 0.90]	0.82 [0.76, 0.86]	0.79 [0.74, 0.86]	0.38 [0.22, 0.52]
	DT	0.43 [0.31, 0.56]	0.47 [0.31, 0.65]	0.56 [0.45, 0.68]	0.39 [0.21, 0.52]
	El.Net	0.95 [0.93, 0.97]	0.89 [0.88, 0.93]	0.71 [0.62, 0.81]	0.37 [0.19, 0.43]
	L.SVM	0.92 [0.86, 0.96]	0.89 [0.86, 0.91]	0.64 [0.54, 0.77]	0.27 [0.16, 0.42]

B1. External Test — Substrate (C vs PM)

		C	PM
Sens	RF	0.95 [0.87, 0.99]	0.45 [0.27, 0.64]
	Ada	0.89 [0.79, 0.96]	0.52 [0.33, 0.70]
	DT	0.77 [0.65, 0.87]	0.68 [0.49, 0.83]
	El.Net	0.83 [0.72, 0.91]	0.52 [0.33, 0.70]
	L.SVM	0.92 [0.83, 0.97]	0.32 [0.17, 0.51]
Spec	RF	0.45 [0.27, 0.64]	0.95 [0.87, 0.99]
	Ada	0.52 [0.33, 0.70]	0.89 [0.79, 0.96]
	DT	0.68 [0.49, 0.83]	0.77 [0.65, 0.87]
	El.Net	0.52 [0.33, 0.70]	0.83 [0.72, 0.91]
	L.SVM	0.32 [0.17, 0.51]	0.92 [0.83, 0.97]
F1	RF	0.86 [0.80, 0.91]	0.58 [0.43, 0.72]
	Ada	0.84 [0.77, 0.90]	0.59 [0.45, 0.72]
	DT	0.80 [0.72, 0.87]	0.63 [0.50, 0.74]
	El.Net	0.81 [0.73, 0.87]	0.55 [0.42, 0.68]
	L.SVM	0.82 [0.75, 0.88]	0.43 [0.29, 0.59]
AUROC	RF	0.84 [0.81, 0.88]	0.84 [0.80, 0.87]
	Ada	0.76 [0.69, 0.82]	0.76 [0.72, 0.78]
	DT	0.71 [0.62, 0.80]	0.72 [0.64, 0.78]
	El.Net	0.76 [0.69, 0.85]	0.74 [0.70, 0.80]
	L.SVM	0.72 [0.67, 0.75]	0.77 [0.64, 0.86]
AUPRC	RF	0.90 [0.88, 0.93]	0.67 [0.58, 0.75]
	Ada	0.84 [0.82, 0.90]	0.57 [0.53, 0.62]
	DT	0.58 [0.45, 0.67]	0.42 [0.33, 0.58]
	El.Net	0.83 [0.79, 0.92]	0.52 [0.48, 0.60]
	L.SVM	0.83 [0.79, 0.85]	0.57 [0.45, 0.70]

B2. External Test — Within-substrate (cascade filter)

		C_{CCW}	C_{CW}	PM_{CCW}	PM_{CW}
Sens	RF	0.86 [0.70, 0.95]	1.00 [0.88, 1.00]	0.89 [0.52, 1.00]	0.40 [0.05, 0.85]
	Ada	0.82 [0.65, 0.93]	0.88 [0.69, 0.97]	0.79 [0.49, 0.95]	0.50 [0.01, 0.99]
	DT	0.85 [0.65, 0.96]	0.72 [0.51, 0.88]	0.92 [0.64, 1.00]	0.62 [0.24, 0.91]
	El.Net	0.90 [0.74, 0.98]	0.92 [0.73, 0.99]	0.70 [0.35, 0.93]	0.50 [0.12, 0.88]
	L.SVM	0.97 [0.84, 1.00]	0.79 [0.60, 0.92]	0.50 [0.12, 0.88]	0.50 [0.07, 0.93]
Spec	RF	1.00 [0.88, 1.00]	0.86 [0.70, 0.95]	0.40 [0.05, 0.85]	0.89 [0.52, 1.00]
	Ada	0.88 [0.69, 0.97]	0.82 [0.65, 0.93]	0.50 [0.01, 0.99]	0.79 [0.49, 0.95]
	DT	0.72 [0.51, 0.88]	0.85 [0.65, 0.96]	0.62 [0.24, 0.91]	0.92 [0.64, 1.00]
	El.Net	0.92 [0.73, 0.99]	0.90 [0.74, 0.98]	0.50 [0.12, 0.88]	0.70 [0.35, 0.93]
	L.SVM	0.79 [0.60, 0.92]	0.97 [0.84, 1.00]	0.50 [0.07, 0.93]	0.50 [0.12, 0.88]
F1	RF	0.92 [0.83, 0.97]	0.92 [0.82, 0.97]	0.80 [0.56, 0.94]	0.50 [0.16, 0.84]
	Ada	0.86 [0.75, 0.93]	0.83 [0.70, 0.92]	0.85 [0.65, 0.96]	0.33 [0.04, 0.78]
	DT	0.80 [0.67, 0.90]	0.77 [0.62, 0.88]	0.86 [0.67, 0.96]	0.71 [0.42, 0.92]
	El.Net	0.92 [0.82, 0.97]	0.90 [0.78, 0.97]	0.70 [0.46, 0.88]	0.50 [0.21, 0.79]
	L.SVM	0.90 [0.80, 0.96]	0.87 [0.75, 0.95]	0.55 [0.23, 0.83]	0.44 [0.14, 0.79]
AUROC	RF	0.90 [0.89, 0.94]	0.92 [0.91, 0.95]	0.70 [0.50, 0.89]	0.76 [0.57, 0.89]
	Ada	0.85 [0.77, 0.88]	0.90 [0.85, 0.95]	0.66 [0.46, 0.78]	0.71 [0.61, 0.90]
	DT	0.88 [0.76, 0.94]	0.87 [0.82, 0.92]	0.59 [0.28, 0.94]	0.64 [0.28, 0.91]
	El.Net	0.95 [0.93, 0.98]	0.95 [0.91, 0.98]	0.60 [0.33, 0.69]	0.63 [0.38, 0.72]
	L.SVM	0.92 [0.89, 0.97]	0.92 [0.88, 0.96]	0.46 [0.08, 0.67]	0.36 [0.00, 0.75]
AUPRC	RF	0.92 [0.91, 0.94]	0.85 [0.80, 0.88]	0.74 [0.52, 0.91]	0.47 [0.17, 0.63]
	Ada	0.85 [0.77, 0.90]	0.82 [0.71, 0.87]	0.78 [0.63, 0.88]	0.38 [0.23, 0.52]
	DT	0.52 [0.26, 0.63]	0.55 [0.29, 0.71]	0.57 [0.37, 0.72]	0.38 [0.21, 0.63]
	El.Net	0.94 [0.91, 0.95]	0.88 [0.84, 0.94]	0.64 [0.49, 0.78]	0.33 [0.18, 0.51]
	L.SVM	0.91 [0.88, 0.94]	0.88 [0.83, 0.93]	0.52 [0.32, 0.73]	0.23 [0.13, 0.63]

TABLE S.X: Definitions of the 30 selected predictors used in the feature-importance analysis (clinical, VCG-derived, and multimodal models).

**Feature (label)**	**Definition**
**Clinical variables**	
BMI	Body-mass index (kg/m^2).
Age	Age in years.
No prior AF	Indicator that the patient has no documented history of atrial fibrillation.
Paroxysmal AF	Indicator of paroxysmal AF history (intermittent, self-terminating episodes).
Persistent AF	Indicator of persistent AF history (sustained episodes requiring intervention or lasting > 7 days per clinical definition).
Sex	Biological sex (female/male).
HTN	Indicator of arterial hypertension.
CHA2DS-VASc	Thromboembolic risk score (higher indicates higher risk).
LVEF	Left-ventricular ejection fraction (%).
**VCG-derived variables**	
Correlation PM CCW	Cosine correlation between the patient's (normalized) atrial VCG loop and the PM counterclockwise (PM CCW) archetype loop.
Correlation PM CW	Cosine correlation between the patient's atrial VCG loop and the PM clockwise (PM CW) archetype.
Correlation C CW	Cosine correlation between the patient's atrial VCG loop and the common clockwise (C CW) archetype.
Correlation C CCW	Cosine correlation between the patient's atrial VCG loop and the common counterclockwise (C CCW) archetype.
Max vector	Maximum dipole magnitude over the atrial cycle, $m_{\max} = \max_i \|\mathbf{r}_i\|_2$.
Complexity	Geometric complexity defined as $P_{\text{VCG}}/P_{\text{ellipse}}$, i.e., loop perimeter divided by the perimeter of a fitted ellipse.
Average homogeneity	Uniformity index of the linear-speed profile defined as $\text{PeakDiff}/\overline{v}$ (higher indicates less uniform speed profile).
Peak differences	Peak-to-peak amplitude of the linear-speed profile: $\max_i v_i - \min_i v_i$.
$\text{Occ}_t^{(\tau)}$	Temporal subthreshold occupancy: fraction of samples with $v_i \leq \tau v_{\max}$ (here $\tau = 0.25$).
$\overline{v}$	Mean linear speed over the full cycle: $\overline{v} = \frac{1}{N-1} \sum_{i=1}^{N-1} v_i$.
$\overline{\omega}$	Mean angular speed over the full cycle: $\overline{\omega} = \frac{1}{N-1} \sum_{i=1}^{N-1} \omega_i$.
AUC_v	Area under the linear-speed profile over the cycle (discrete integral), e.g. $\sum_i v_i \, \Delta t$.
AUC_ω	Area under the angular-velocity profile over the cycle (discrete integral), e.g. $\sum_i \omega_i \, \Delta t$.
Kurtosis (linear)	Kurtosis of the linear-speed distribution $\{v_i\}$ (profile sharpness/heavy tails).
Angular kurtosis	Kurtosis of the angular-speed distribution $\{\omega_i\}$.
Linear velocity 1st segment	Mean linear speed computed over the first predefined segment of the cycle.
Linear velocity 2nd segment	Mean linear speed computed over the second predefined segment of the cycle.
Angular velocity 1st segment	Mean angular speed computed over the first predefined segment of the cycle.
Angular velocity 2nd segment	Mean angular speed computed over the second predefined segment of the cycle.
Fastest direction of VCG	Direction (angle/axis) at which the instantaneous linear speed attains its maximum; derived from the velocity vector at $i^* = \arg\max_i v_i$.
Sign of fastest direction	Sign of the fastest-direction component.
